# Exploring estrogen-related mechanisms in ovarian carcinogenesis: association between bone mineral density and ovarian cancer risk in a multivariable Mendelian randomization study

**DOI:** 10.1007/s10552-024-01926-9

**Published:** 2024-10-18

**Authors:** Karen M. Tuesley, Penelope M. Webb, Melinda M. Protani, Peter Donovan, Susan J. Jordan, Suzanne Dixon-Suen

**Affiliations:** 1 School of Public Health, Faculty of Medicine, The University of Queensland, Brisbane, Australia; 2 Population Health Program, QIMR Berghofer Medical Research Institute, Brisbane, Australia; 3 Clinical Pharmacology Department, Royal Brisbane and Women’s Hospital, Brisbane, Australia; 4 Faculty of Medicine, The University of Queensland, Brisbane, Australia; 5 Cancer Epidemiology Division, Cancer Council Victoria, Melbourne, Australia; 6 Institute for Physical Activity and Nutrition, Deakin University, Geelong, VIC Australia

**Keywords:** Epithelial ovarian cancer, Bone mineral density, Mendelian randomization, Oestrogen

## Abstract

**Background:**

Estrogen may play a role in epithelial ovarian cancer (EOC) carcinogenesis, with effects varying by EOC histotype. Measuring women’s long-term exposure to estrogen is difficult, but bone mineral density (BMD) may be a reasonable proxy of longer-term exposure. We examined this relationship by assessing the association between genetic predisposition for higher BMD and risk of EOC by histotype.

**Methods:**

We used Mendelian randomization (MR) to assess associations between genetic markers for femoral neck and lumbar spine BMD and each EOC histotype. We used multivariable MR (MVMR) to adjust for probable pleiotropic traits, including body mass index, height, menarcheal age, menopausal age, smoking, alcohol intake, and vitamin D.

**Results:**

Univariable analyses suggested greater BMD was associated with increased risk of endometrioid EOC (per standard deviation increase; lumbar spine OR = 1.21; 95% CI 0.93,1.57, femoral neck: OR = 1.25; 0.99,1.57), but sensitivity analyses indicated that pleiotropy was likely. Adjustment using MVMR reduced the magnitude of estimates slightly (lumbar spine: OR = 1.13; 95% CI 1.00,1.28, femoral neck: OR = 1.18; 1.03,1.36). Results for lumbar spine BMD and high-grade serous EOC were also suggestive of an association (univariable MR: OR = 1.16; 95% CI 1.03,1.30; MVMR: OR = 1.06; 0.99,1.14).

**Conclusion:**

Our study found associations between genetic predisposition to higher BMD, a proxy for long-term estrogen exposure, and risk of developing endometroid and high-grade serous EOC cancers. These findings add to existing evidence of the relationship between estrogen and increased risk of EOC for certain histotypes.

**Supplementary Information:**

The online version contains supplementary material available at 10.1007/s10552-024-01926-9.

## Introduction

Epithelial ovarian cancer (EOC) is the eighth most common cancer in women worldwide and, as five-year survival remains at less than 50% [1], understanding the mechanisms driving cancer development is important to facilitate prevention [2]. Reproductive factors have the well-established relationships with EOC but the mechanism by which these influence risk is not clear, although direct effects of sex hormones have long been proposed. In particular, some evidence, although not all, suggests that estrogen exposure may be positively associated with EOC carcinogenesis [3, 4], with the effects varying by EOC histotype [5]. Estrogen receptors are present in the majority of serous and endometrioid EOC cancers [5] and menopausal hormone therapy (MHT) use has also been linked to an increased risk of serous and endometrioid EOC [6] suggesting that estrogen plays a role in the development of these cancers. However, directly measuring women’s long-term exposure to estrogen is difficult given cyclical and temporal variations in estrogen levels.

One alternative to traditional observational studies for studying etiological relationships is Mendelian randomization (MR). Using genetic variation to explore causal relationships, MR studies can minimize environmental influences that may bias the association [7], provide a stable measure of long-term exposure, and allow exploration of associations for rare outcomes, such as EOC, including its histotypes [8], without the need to measure exposure directly in the cohort at risk. MR studies replace the exposure of interest with genetic variants, usually single-nucleotide polymorphisms (SNPs), which predict that exposure, and assess the relationship between these SNPs and the outcome of interest. While using SNPs associated with long-term estrogen exposure would be ideal to investigate the relationship between estrogen and EOC, few genome-wide association studies (GWAS) have examined levels of oestrogens (such as oestradiol) in females [9, 10]. The largest, identified two genetic variants associated with oestradiol at genome-wide significance (*p* < 5 × 10^−8^) in women, but did not account for menstrual cycle phase (in premenopausal women) or years since menopause (in postmenopausal women), factors which heavily influence oestradiol level and complicate GWAS of this exposure.

One potentially useful proxy for long-term estrogen exposure is bone mineral density (BMD), which is known to be strongly related to pre- and postmenopausal estrogen levels, with lower estrogen levels associated with lower BMD [11, 12]. Therefore, genetic variation in BMD may be a reasonable surrogate of long-term estrogen exposure [13]. Large GWAS have identified SNPs associated with either femoral neck or lumbar spine BMD, which are both strongly related to osteoporotic fracture [14, 15]. While our understanding of the complex pathways and connections between all these loci is still in its infancy, some studies have identified relationships between these loci and estrogen pathways [16–20]. In addition, BMD has been used as a proxy for long-term estrogen exposure in etiological studies of other estrogen-related cancers, such as breast and endometrial cancers [21–23] but, to our knowledge, no studies have assessed the association between BMD and risk of ovarian cancer. One study investigated the association between bone fractures (a consequence of low BMD) and EOC, but, noting the small number of case women (*n* = 151), it did not find any associations and was not able to assess associations by histological subtype [24].

We used MR to assess the association between genetic predisposition for high femoral neck or lumbar spine BMD and risk of the main EOC histotypes (high-grade serous, low-grade serous, mucinous, endometrioid, and clear cell) to shed light on the relationship between estrogen and ovarian cancer carcinogenesis. We hypothesized that higher BMD at the lumbar spine or femoral neck (as a surrogate for higher overall estrogen exposure) would be associated with increased risk of EOC, specifically the serous and endometrioid histotypes.

## Methods

### Instrumental variables: Obtaining genetic associations

**Fig. 1:**
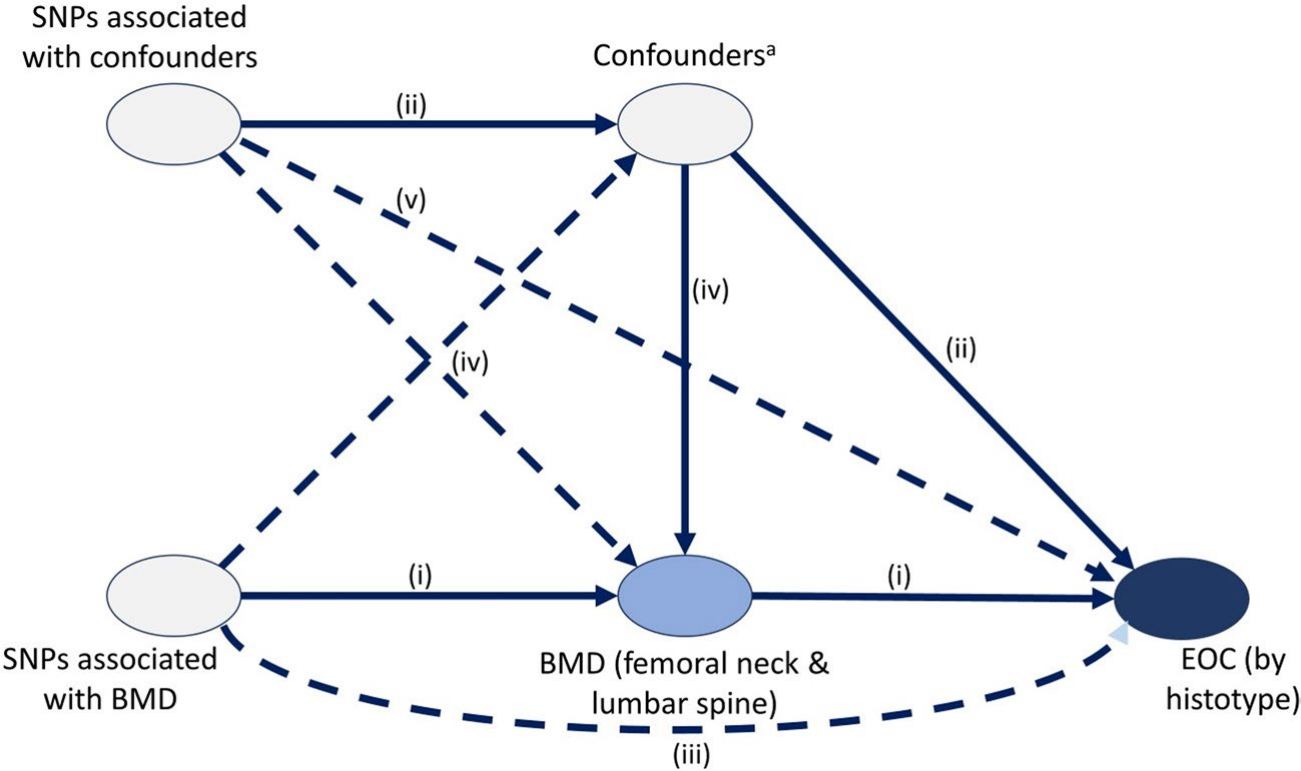
Directed acyclic graph for the association between bone mineral density and epithelial ovarian cancer. *BMD* bone mineral density, *EOC* epithelial ovarian cancer, *MR* Mendelian randomization, *MVMR*: multivariable Mendelian randomization, *SNPs* single-nucleotide polymorphisms. Dashed lines represent associations that would violate assumptions (b) and (c) Pathways tested in the main analyses: MR-IVW: direct pathway (i); MVMR-IVW: direct (i) and confounder pathways (ii, iv); MR-Egger: as per MR-IVW but also includes pathway from SNPs to outcome (iii); MRMV-Egger; as per MVMR-IVW but also includes the direct pathways from SNPs to outcome (iii, v). ^a^Confounders include age at menarche, age at menopause, adult attained height, body mass index, smoking, vitamin D and alcohol intake frequency

For MR, a genetic variant is a valid instrument if it meets the following criteria: (a) the variant is predictive of the exposure; (b) the variant is independent of any confounding factors of the exposure-outcome association; and (c) the variant is conditionally independent of the outcome given the exposure and the confounding factors [25, 26]. Horizontal pleiotropy occurs when a gene influences more than one trait, and this pleiotropy can lead to biased results in MR analyses if the additional trait is on a different causal pathway to the outcome [27]. Given the complex genetic underpinnings of many traits, there is a possibility that SNPs for BMD may also influence other risk factors for EOC, which, similar to confounding in standard epidemiological studies, might bias MR results. Examples include smoking, body mass index (BMI), age at menopause, age at menarche, height, alcohol intake, and vitamin D [2, 28–30]. Multivariable MR (MVMR) allows us to fit genetic instruments for multiple risk factors in a single model [27], accounting to some extent for possible pleiotropy. For example, this approach was used in a recent study to investigate the BMI-independent causal pathway from BMD to osteoarthritis risk [31]. Figure 1 [32] shows the directed acyclic graph depicting the causal assumptions underpinning our analysis.

We used summary-level data on genetic associations for each exposure, outcome, and potential confounder available through the University of Bristol MRC Integrative Epidemiology Unit OpenGWAS database[33] from the Genetic Factors for Osteoporosis Consortium (GEFOS) [14], UK Biobank [34, 35], and the Ovarian Cancer Association Consortium (OCAC) [8] (summarized in Table 1). We used the TwoSampleMR[33, 36] package in R [37] which takes estimates directly from OpenGWAS, to select genetic instruments (i.e., SNPs) with a significance level of *p* < 5 × 10^−8^ (widely accepted and conservative measure of association for genetic variants)[38] for each exposure to include in the analysis. For the MVMR analysis, we performed additional extractions to obtain estimates for instruments associated with the potential confounders. Where data on the estimates for a selected instrument and an outcome were missing, the TwoSampleMR package allowed proxy SNPs with a correlation coefficient with the BMD SNP equal to or above 0.8. Palindromic SNPs, which have the same alleles on the forward and reverse strands (A/T or C/G), were removed if the trait-increasing allele could not be determined from allele frequency (between 0.3 and 0.7).

**Table 1:** Instruments and sources for exposure, confounding and outcome variables

Variable description	Variable id:	Publication year	Measurement	Population ethnicity	Sex	Sample size	Associated SNPs^a^	SNPs included in analyses^b^	*F*-statistic [40]
Exposures									
Lumbar spine bone mineral density	ieu-a-982^c^	2015	Continuous (g/cm^2^)	European	M/F	28,498	24	21^f,g^	46.3
Femoral neck bone mineral density	ieu-a-980^c^	2015	Continuous (g/cm^2^)	European	M/F	32,735	21	20^g^	52.7
Confounders									
Standing height	ukb-b-10787^d^	2018	Continuous	European	M/F	461,950	773	655	129.9
Age at menopause (last menstrual period)	ukb-b-17422^d^	2018	Integer, years	European	F	143,819	114	88	77.1
Body mass index (BMI)	ukb-b-19953^d^	2018	Continuous	European	M/F	461,460	458	389	61.2
Age when periods started (menarche)	ukb-b-3768^d^	2018	Integer, years	European	F	243,944	200	177	65.3
Alcohol intake frequency	ukb-b-5779^d^	2018	Categorical ordered	European	M/F	462,346	99	84	45.9
Pack years adult smoking as proportion of life span exposed to smoking	ukb-b-7460^d^	2018	Continuous, units	European	M/F	142,387	14	14	69.6
Vitamin D	ukb-d-30890_irnt^d^	2018	Continuous, nmol/L	European	M/F	449,913	59	50	216.1
Outcomes									
High-grade serous ovarian cancer	ieu-a-1121^e^	2017	Binary	European	F	53,978 (13,037 cases)	n/a^h^	n/a^h^	n/a^h^
Low-grade serous ovarian cancer	ieu-a-1122^e^	2017	Binary	European	F	41,953 (1,012 cases)	n/a^h^	n/a^h^	n/a^h^
Endometrioid ovarian cancer	ieu-a-1125^e^	2017	Binary	European	F	43,751 (2,810 cases)	n/a^h^	n/a^h^	n/a^h^
Clear-cell ovarian cancer	ieu-a-1124^e^	2017	Binary	European	F	42,307 (1,366 cases)	n/a^h^	n/a^h^	n/a^h^
Invasive mucinous ovarian cancer	ieu-a-1123^e^	2017	Binary	European	F	42,358 (1,417 cases)	n/a^h^	n/a^h^	n/a^h^
Other									
Bioavailable testosterone	ieu-b-4869^d^	2020	Continuous	European	F	180,386	115	101	71.1

^a^SNPs associated to significance level of *p* < 5 × 10^−8^

^b^Where associations for SNPs were not available for the outcome variables, proxies were used if the correlation coefficient was equal or higher than 0.8. If there were no proxies available, the SNP was not included in the analyses. Palindromic SNPs with allele frequency between 0.3 and 0.7 were excluded. We additionally excluded SNPs associated with more than one trait, including 6 SNPs for BMI, 5 for standing height, and 1 for age at menarche

^c^Published by the Genetic Factors for Osteoporosis Consortium (GEFOS) [14]

^d^Published by UK Biobank. ukb-b/ieu-b: MRC IEU[34] and ukb-d: Neale lab round 2 release 2018 [35]

^e^Published by the Ovarian Cancer Association Consortium (OCAC) [8]

^f^Two palindromic SNPs associated with lumbar spine bone mineral density excluded from analysis

^g^One SNP associated with each of lumbar spine bone mineral density and femoral neck bone mineral density excluded as there was no association and no proxy available for the outcome variables

^h^Outcome measure only

We included 21 SNPs for lumbar spine BMD and 20 SNPs for femoral neck BMD, as well as 1762 SNPs associated with potential confounding variables (Table 1). We excluded six SNPs which were associated at *p* < 5 × 10^−8^ with more than one trait (five associated with both BMI and standing height, and one associated with both BMI and age at menarche). We additionally excluded two SNPs which were associated with high-grade serous EOC (one each for BMI and standing height), from the analyses with high-grade serous EOC as an outcome. We oriented the genetic variants for BMD so the effect allele we analyzed/reported results for had a positive association with BMD (i.e., one standard deviation (SD) increase in lumbar spine or femoral neck BMD). We assessed the linkage disequilibrium between SNPs associated with oestradiol in females and BMD SNPs (Supplementary Methods, Supplementary Table 1).

In a supplementary analysis [32], we explored whether other sex hormones may contribute to any associations found between BMD and EOC. The relationship between testosterone and BMD in females is not well understood, although there is some evidence of a positive association [39]. We therefore conducted a sensitivity analysis to include bioavailable testosterone in females as a covariate in the MVMR analysis.

### Statistical analysis

We first conducted two-sample univariable MR analyses for the association between the BMD measures (lumbar spine and femoral neck) and each EOC histotype using the inverse-variance weighted (IVW) method. As the IVW method is sensitive to outliers and directional pleiotropy, we performed several sensitivity analyses. To assess the influence of outliers or invalid instruments, we used Cochran’s Q test (IVW) and Rücker’s Q test (MR-Egger) to assess heterogeneity. We assessed instrument strength using the *F*-statistic [40]. We used “leave-one-out” sensitivity analysis, which produces IVW estimates after omitting each SNP in turn, to determine whether a single SNP was strongly influencing the association. We used MR-Egger which provides a test for pleiotropy effects based on deviation of the regression intercept from the origin and provides an estimate of causal association using the slope coefficient [41]. We also used weighted median method which provides a consistent estimate even if up to 50% of instruments are invalid [25] and the Iterative Mendelian Randomisation and Pleiotropy (IMRP) approach removing variants with horizontal pleiotropy [42]. Lastly, we used the MR Robust Adjusted Profile Score (MR-RAPS) which is robust to weak instruments[43] and a flexible normal-mixture models (MR-Mix) which is robust in the presence of unobserved confounding factors [44].

For the MVMR analysis, we additionally included SNPs associated with standing height, age at menopause, BMI, age at menarche, alcohol intake frequency, smoking, and vitamin D. We first included each covariate separately in the model, then included all covariates. We used the inverse-variance weighted method for our primary analyses, and sensitivity analyses to evaluate the potential for residual pleiotropy and invalid instruments using MVMR-Egger and multivariable-weighted median MR (MVMR-Median) methods. As an additional sensitivity analysis, we used the MVMR-Lasso method which detects and removes likely invalid instruments and performs well with moderate to high levels of pleiotropy [27]. We used the Mendelian Randomization[27] R[37] packages in RStudio [45] for the MVMR analyses.

We estimated odds ratios (ORs) and 95% confidence intervals (CI) for the relationship between each exposure trait and risk of each EOC histotype. We reported our findings in accordance with the MR [46] and STROBE-MR guidelines [47].

## Results

**Fig. 2:**
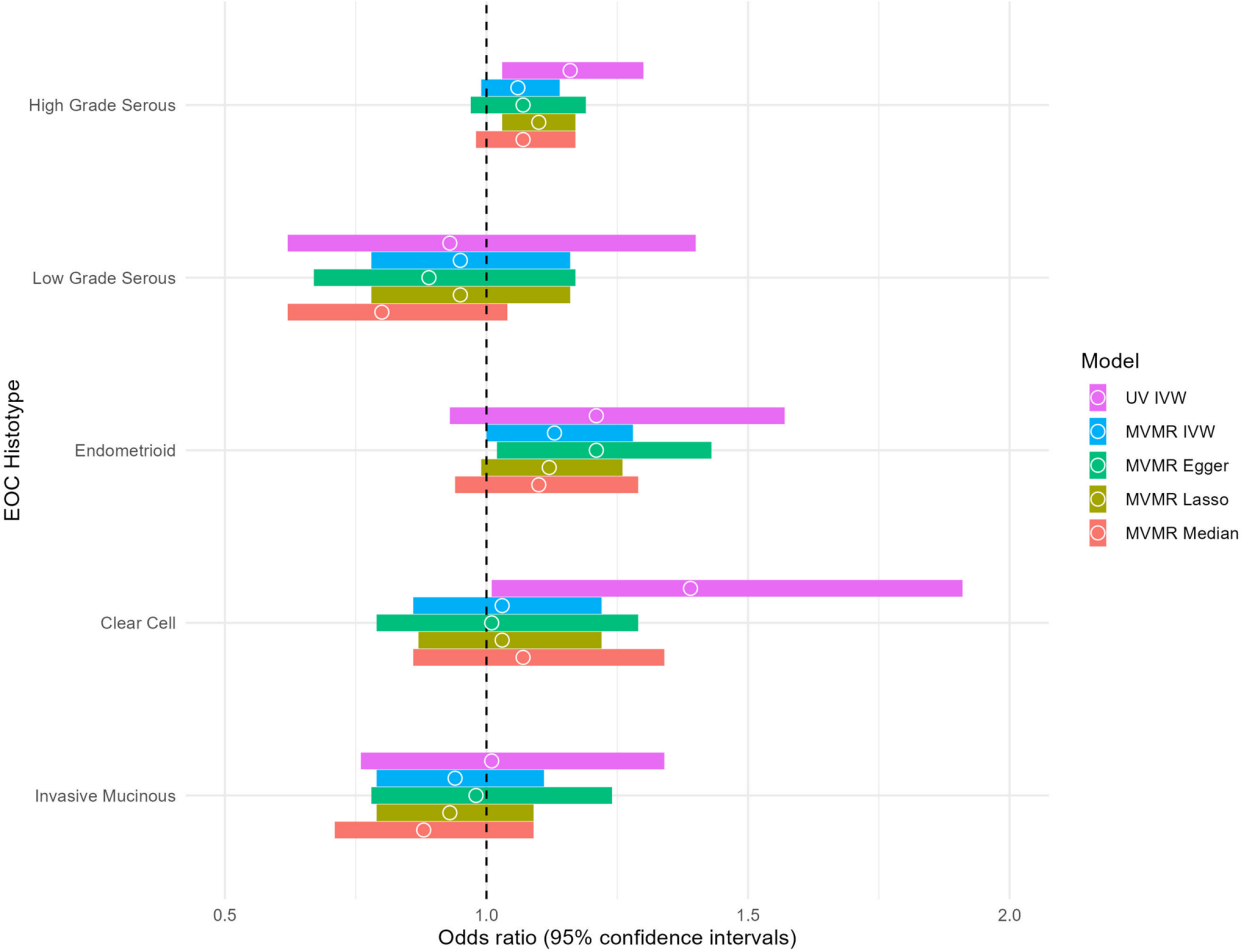
Comparison between univariable and multivariable Mendelian randomization results for the association between lumbar spine bone mineral density and each epithelial ovarian cancer histotype. *IVW* inverse-variance weighted, *MVMR* multivariable Mendelian randomization, *UV* univariable. Multivariable Mendelian randomization model adjusted for body mass index, standing height, alcohol intake frequency, smoking (pack years as proportion of life span exposed to smoking), vitamin D, age at menopause, and age at menarche

**Fig. 3:**
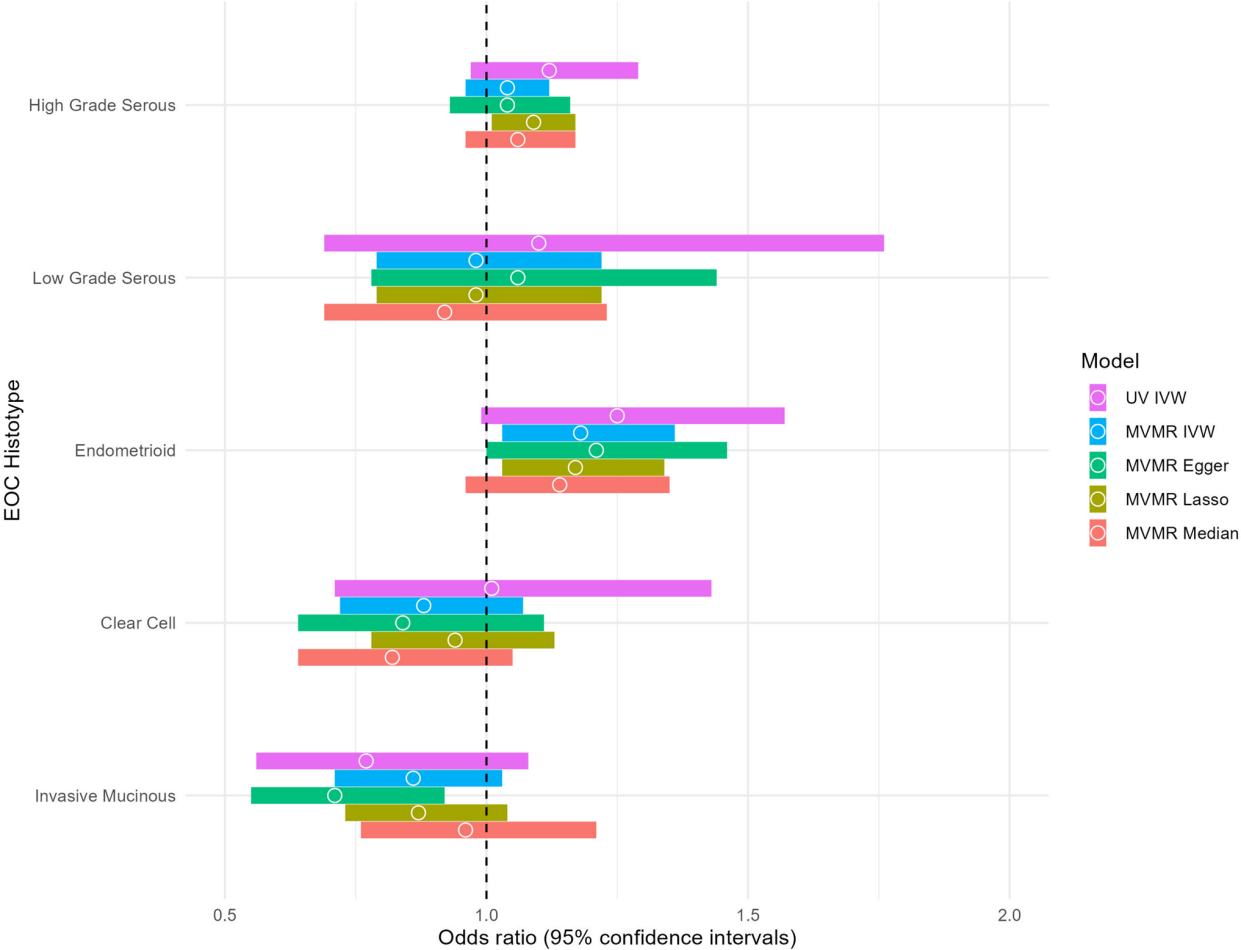
Comparison between univariable and multivariable Mendelian randomization results for the association between femoral neck bone mineral density and each epithelial ovarian cancer histotype. *IVW* inverse-variance weighted, *MVMR* multivariable Mendelian randomization, *UV* univariable. Multivariable Mendelian randomization model adjusted for body mass index, standing height, alcohol intake frequency, smoking (pack years as proportion of life span exposed to smoking), vitamin D, age at menopause, and age at menarche

The main results are shown in Table 2 and Figs. 2–3. The *F*-statistic for each exposure and covariate indicated that weak instrument bias was unlikely and results for MR-RAPS were consistent with MR-IVW results for all outcomes. At least one BMD SNP had moderate to high linkage disequilibrium for each estradiol-associated SNP (Supplementary Materials, Supplementary Table 1). Supplementary Table 3 [32] shows the results for adding bioavailable testosterone to the MVMR model and for adding each covariate separately. Forest plots and scatter plots for each univariable MR analysis are shown in Supplementary Figs. 1 to 10 [32].

**Table 2:** Results for univariable and multivariable Mendelian randomization analyses for the association between lumbar spine and femoral neck bone mineral density and risk of epithelial ovarian cancer by histotype

Method	High-grade serous		Low-grade serous		Endometrioid		Clear cell		Invasive mucinous	
	OR (95% CI)	*p*^a^	OR (95% CI)	*p*^a^	OR (95% CI)	*p*^a^	OR (95% CI)	*p*^a^	OR (95% CI)	*p*^a^
Lumbar spine BMD										
Univariable MR-IVW	1.16 (1.03, 1.30)		0.93 (0.62, 1.40)		1.21 (0.93, 1.57)b		1.39 (1.01, 1.91)		1.01 (0.76, 1.34)	
Multivariable MR-IVW	1.06 (0.99, 1.14)^b^		0.95 (0.78, 1.16)		1.13 (1.00, 1.28)^b^		1.03 (0.86, 1.22)^b^		0.94 (0.79, 1.11)	
Sensitivity analyses										
MR-Egger	1.02 (0.63, 1.64)	0.58	4.22 (0.88, 20.3)	0.07	1.60 (0.55, 4.66)^b^	0.60	2.27 (0.61, 8.38)	0.46	2.14 (0.69, 6.62)	0.20
MR-WM	1.17 (0.99, 1.39)		1.03 (0.61, 1.73)		1.13 (0.85, 1.50)		1.45 (0.94, 2.23)		1.09 (0.73, 1.64)	
IMRP	1.20 (1.07, 1.34)		1.03 (0.72, 1.47)		1.33 (1.05, 1.68)		1.31 (0.97, 1.77)		1.07 (0.70, 1.25)	
MR-RAPS	1.16 (1.04, 1.31)		0.92 (0.65, 1.31)		1.22 (0.99, 1.50)		1.40 (1.04, 1.88)		1.01 (0.76, 1.35)	
MR-MIX	1.06 (0.99, 1.14)		1.03 (0.92, 1.14)		1.00 (0.89, 1.13)		1.00 (0.84, 1.20)		1.01 (0.94, 1.07)	
MVMR-Egger	1.07 (0.97, 1.19)^b^		0.89 (0.67, 1.17)		1.21 (1.02, 1.43)^b^		1.01 (0.79, 1.29)^b^		0.98 (0.78, 1.24)	
MVMR-Lasso	1.10 (1.03, 1.17)		0.95 (0.78, 1.16)		1.12 (0.99, 1.26)		1.03 (0.87, 1.22)		0.93 (0.79, 1.09)	
MVMR-Median	1.07 (0.98, 1.17)		0.80 (0.62, 1.04)		1.10 (0.94, 1.29)		1.07 (0.86, 1.34)		0.88 (0.71, 1.09)	
Femoral neck BMD										
Univariable MR-IVW	1.12 (0.97, 1.29)		1.10 (0.69, 1.76)		1.25 (0.99, 1.57)		1.01 (0.71, 1.43)		0.77 (0.56, 1.08)	
Multivariable MR-IVW	1.04 (0.96, 1.12)^b^		0.98 (0.79, 1.22)		1.18 (1.03, 1.36)^b^		0.88 (0.72, 1.07)^b^		0.86 (0.71, 1.03)	
Sensitivity analyses										
MR-Egger	1.05 (0.49, 2.24)	0.86	1.80 (0.14, 23.0)	0.70	0.75 (0.22, 2.57)	0.42	0.61 (0.09, 4.06)	0.60	1.80 (0.31, 10.3)	0.34
MR-WM	1.11 (0.91, 1.34)		0.86 (0.48, 1.53)		1.11 (0.81, 1.52)		0.65 (0.41, 1.02)		0.79 (0.51, 1.23)	
IMRP	1.09 (0.96, 1.24)		1.12 (0.75, 1.68)		^c^		^c^		0.83 (0.60, 1.15)	
MR-RAPS	1.12 (0.99, 1.28)		1.10 (0.74, 1.64)		1.25 (0.98, 1.59)		1.00 (0.72, 1.40)		0.77 (0.55, 1.07)	
MR-MIX	1.00 (0.89, 1.12)		1.06 (0.53, 2.11)		1.06 (1.00, 1.12)		0.91 (0.80, 1.04)		0.96 (0.87, 1.05)	
MVMR-Egger	1.04 (0.93, 1.16)^b^		1.06 (0.78, 1.44)		1.21 (1.00, 1.46)^b^		0.84 (0.64, 1.11)^b^		0.71 (0.55, 0.92)	
MVMR-Lasso	1.09 (1.01, 1.17)		0.98 (0.79, 1.22)		1.17 (1.03, 1.34)		0.94 (0.78, 1.13)		0.87 (0.73, 1.04)	
MVMR-Median	1.06 (0.96, 1.17)		0.92 (0.69, 1.23)		1.14 (0.96, 1.35)		0.82 (0.64, 1.05)		0.96 (0.76, 1.21)	

*BMD* bone mineral density, *CI* confidence interval, *IMRP* iterative Mendelian randomization and pleiotropy, *IVW* inverse variance weighted, *MR* Mendelian randomization, *MIX* flexible normal-mixture model, *MVMR* multivariable Mendelian randomization, *OR* odds ratio, *P* P-value, *RAPS* robust adjusted profile score, *WM* weighted median

^a^*p*-value for the MR-Egger intercept

^b^Test of heterogeneity *P* < 0.05 (Cochran’s Q for IVW and Rücker’s Q for MR-Egger)

^c^There were no pleiotropic outliers for the IMRP analysis

### High-grade serous EOC

The univariable MR-IVW model showed that each standard deviation (SD) increase in lumbar spine BMD was associated with a 16% increased risk of high-grade serous EOC (1 SD = 0.62 g/cm^2^, OR = 1.16, 95% CI: 1.03,1.30). The estimates for femoral neck BMD also suggested a possible association (1 SD = 0.41 g/cm^2^, OR = 1.12, 95% CI: 0.97,1.29). The results of sensitivity analyses applying different univariable MR methods were mixed. The weighted median and IMRP MR results were consistent with the MR-IVW. However, MR-Egger estimates were closer to the null (OR = 1.02, 95%CI:0.63,1.64 and OR = 1.05, 95% CI: 0.49,2.24 for lumbar spine and femoral neck BMD, respectively), with minor asymmetry in the funnel plots of instrument strength versus MR estimates (Supplementary Fig. 1d) [32]. MR-Mix estimates were also closer to the null, suggesting the possibility of directional pleiotropy even though the MR-Egger intercepts were not significantly different from zero (*p* = 0.6 and 0.9 for lumbar spine and femoral neck BMD, respectively). Supplementary Fig. 1c shows the reduction in the effect size for the MR-Egger estimates for lumbar spine BMD when allowing for intercept to deviate from the origin. Heterogeneity of MR estimates across the SNPs for either exposure was not suggested by Cochran’s Q test, nor by analyses by SNP or omitting each SNP in turn (Supplementary Figs. 1a,b, 2a,b) [32].

Figures 2 and 3 show a comparison of the MR-IVW results with the four MVMR models when we included SNPs associated with potential confounders. Although the results for MVMR-IVW were attenuated toward the null (lumbar spine BMD: OR = 1.06, 95% CI: 0.99,1.14 and femoral neck BMD: OR = 1.04, 95% CI: 0.95,1.12, respectively), Cochran’s, and Rücker’s Q tests suggested heterogeneity of estimates across SNPs. After removing outliers (Supplementary Table 2), MR-Lasso indicated a slightly stronger associations for both exposures (lumbar spine BMD: OR = 1.10, 95%CI: 1.03,1.17 and femoral neck BMD: OR = 1.09, 95% CI: 1.01,1.17, respectively). Results were reasonably consistent across all MVMR models for both BMD measures. Our supplementary analysis including bioavailable testosterone in the MVMR model (Supplementary Table 3) [32] did not change the results.

### Low-grade serous EOC

Neither the univariable nor the MVMR-IVW analyses showed an association between lumbar spine or femoral neck BMD and risk of low-grade serous EOC (MVMR-IVW: OR = 0.95, 95% CI: 0.78,1.16 and OR = 0.98, 95% CI: 0.79,1.22, respectively). Results of sensitivity analyses were generally consistent with this.

### Endometrioid EOC

The univariable analyses for both lumbar spine and femoral neck BMD suggested an increased risk of endometrioid EOC (OR = 1.21, 95% CI: 0.93,1.57 and OR = 1.25, 95% CI: 0.99,1.57 respectively). For lumbar spine BMD, Cochran’s Q test indicated heterogeneity in the estimates across instruments; however, the results were similar to femoral neck BMD, which did not show heterogeneity. The results of sensitivity analyses applying different univariable methods were mixed. The weighted median MR results, although attenuated, were consistent with the MR-IVW. However, the MR-Egger and attenuated MR-Mix estimates indicated that directional pleiotropy was possible and therefore supporting proceeding to an MVMR approach.

The MVMR-IVW estimates were slightly attenuated, however still indicated an increased risk (OR = 1.13, 95% CI: 1.00,1.28 and OR = 1.18, 95% CI: 1.03,1.36 for lumbar spine and femoral neck BMD, respectively). Figures 2 and 3 show that the results were consistent across the MVMR sensitivity analyses, although Cochran’s and Rücker’s Q tests for both exposures (Table 2) suggested heterogeneity of MVMR-IVW estimates across SNPs. After removing outliers, MR-Lasso suggested an increase in risk for both exposures (OR = 1.12, 95% CI: 0.99,1.26 and OR = 1.17, 95% CI: 1.03,1.34, respectively), although the association was of slightly weaker magnitude for lumbar spine BMD. Including bioavailable testosterone in the MVMR model did not material change the results (Supplementary Table 3) [32].

### Clear-cell EOC

In the univariable analysis, lumbar spine BMD was associated with an increase in risk of clear cell EOC (OR = 1.39, 95% CI: 1.01,1.91); however, there was a lack of concordance from MR-Egger and some asymmetry in the funnel plot of instrument strength versus MR estimates (Supplementary Fig. 7)[32] consistent with the possibility of influence by other traits via directional pleiotropy. There was no association after adjusting for possible confounders in the MVMR-IVW analysis (OR = 1.03, 95% CI: 0.86,1.22), with results consistent across MVMR methods including MR-Lasso.

In addition, there was no association in the univariable analysis for femoral neck BMD (OR = 1.01, 95% CI: 0.71,1.43), and the association became inverse in the MVMR-IVW analysis (OR = 0.88, 95% CI: 0.72,1.07). MVMR sensitivity analyses were consistent with MVMR-IVW results; although Cochran’s and Rücker’s Q tests suggested heterogeneity of MVMR estimates across SNPs and after removing outliers, MR-Lasso results were attenuated and remained unsupportive of an association (OR = 0.94, 95% CI: 0.78,1.13).

### Invasive mucinous EOC

The univariable and multivariable analyses did not show an association between lumbar spine BMD and invasive mucinous EOC (OR = 1.01, 95% CI: 0.76,1.34 and OR = 0.94, 95% CI: 0.79,1.11, respectively). Discordance of IVW versus MR-Egger results seen in univariable models were resolved in multivariable models. Results were consistent across MVMR methods including MR-Lasso.

The univariable analysis for femoral neck BMD suggested an association with a reduced risk of invasive mucinous EOC (OR = 0.77, 95% CI: 0.56, 1.08), however the MR-Egger sensitivity analysis, with a reversed direction of association and some asymmetry in the funnel plot of instrument strength versus the MR estimates suggested that this result may be due to pleiotropy (Supplementary Fig. 10) [32]. In the MVMR-IVW analysis, the results attenuated toward the null, however still were suggestive of an association with a reduced risk of mucinous EOC (OR = 0.86, 95% CI: 0.71, 1.03). While the MR-Lasso result after excluding outliers (OR = 0.87, 95% CI: 0.73, 1.04) was virtually identical, there were inconsistent results across the other MVMR sensitivity analyses, with MVMR-Egger finding a risk reduction of 29% while the MVMR-Median point estimate was close to null, suggesting that there may be some residual bias due to pleiotropy for the association between femoral neck BMD and invasive mucinous EOC.

## Discussion

We used multivariable MR to assess the association between lumbar spine and femoral neck BMD, as proxy measures of long-term exposure to estrogen and five EOC histotypes, after accounting for potentially pleiotropic traits. We found that each standard deviation increase in lumbar spine (0.62 g/cm^2^) or femoral neck (0.41 g/cm^2^) BMD was associated with an increased risk of high-grade serous EOC (6% and 4%, respectively) and endometrioid EOC (13% and 18%, respectively).

The direction of our findings is supported by the literature on the relationship between estrogen and EOC histotypes. A large pooled analysis of 21 observational studies found use of menopausal hormone therapy to be associated with an increased risk of endometroid and serous EOC [2]. The same study found early age at menopause to be associated with a decreased risk of serous, endometrioid, and clear-cell EOC [2]. While estrogen receptors, commonly found in endometrioid EOC, have been shown to stimulate cell proliferation in cells containing estrogen receptors [5], the role estrogen plays in EOC development is unclear [48]. Our MR analysis explored the association between a proxy for long-term estrogen exposure and EOC, without the influence of environmental influences, such as hormone use. Our multivariable MR analysis found associations for this exposure independently of other known risk factors for EOC, such as age at menopause and height.

Our study had several strengths. This was the first study to use MR to assess the relationship between BMD, as a stable proxy for estrogen, and EOC, overcoming biases potentially present in prior observational studies. We report results by EOC histotype, which is important due to evidence of their differing etiology. We performed two sets of analyses, one for each of lumbar spine and femoral neck BMD, which allowed us to show consistency in the associations across the two BMD measures and minimized the likelihood of chance findings, although there was some overlap in SNPs. We used the genetic association data from the largest GWAS available, increasing the MR estimate precision. We used multivariable MR analyses to account for pleiotropy by other traits that have a relationship with EOC and BMD. For all analyses of BMD measures and EOC histotypes other than the relationship between femoral neck BMD and clear cell EOC, the results were attenuated toward the null in the multivariable MR models, demonstrating the importance of considering genetic confounders in this study. Where primary (IVW) multivariable MR analyses showed heterogeneity across the variants, we prioritized results from MR-Lasso (which removes outlying SNPs) and used other sensitivity analyses to evaluate the potential for directional pleiotropy and invalid instruments and found that these were unlikely to have a material effect on our results.

Our study also had several limitations. MR studies require large sample sizes to achieve comparable levels of statistical power to observational studies. Therefore, while we did not find associations for EOC histotypes other than high-grade serous and endometrioid, this could be due to the limited statistical power of MR studies. We used BMD as a proxy for estrogen as we were not able to use a direct measure of long-term estrogen exposure in our analysis. There is also a lack of sex-specific GWAS estimates, therefore our exposure and confounding variables were not specific to females. While BMD is not a perfect measure for estrogen, it appears to be a reasonable surrogate, with moderate to high linkage disequilibrium with SNPs associated with oestradiol levels in females. There is a well-established link between BMD and estrogen and recent experimental data have shown links between loci for BMD and estrogen signaling [16–19]. Our supplementary analysis including bioavailable testosterone in females showed that it is unlikely to be contributing to the association [32]. Family and twin studies have found both lumbar spine and femoral neck BMD to be highly heritable; however, genetic variants identified to date explain only 2–6% of the variation in BMD [49, 50]. As larger studies are performed, more genetic variation may be explained [15] and this may improve the power of similar MR studies in future. Using two-sample MR, we were also reliant on published association between the BMD SNPs and EOC histotypes being available. There were three SNPs predicting lumbar spine BMD and one predicting femoral neck BMD that we were unable to include as instruments in our analysis, as there were no data available on their association with EOC even after considering proxies. There was also potential for some heterogeneity as the GWAS used for the exposures and most confounders were not restricted to women. Despite these limitations, we were able to detect an association between both femoral neck and lumbar spine BMD and risk of high-grade serous and endometrioid EOC cancers in our multivariable MR models.

While further investigation is required, our study found associations between genetic predisposition to BMD, a proxy for long-term estrogen exposure, and risk of developing high-grade serous and endometroid EOC cancers, and these associations persisted after accounting for confounding traits in multivariable MR analyses. These findings add to existing evidence of the relationship between estrogen and increased risk of EOC only for certain histotypes.

## Supplementary Information

Below is the link to the electronic supplementary material.

Supplementary file1 (PDF 817 KB)

## Data Availability

The datasets were derived from sources in the public domain: the University of Bristol MRC Integrative Epidemiology Unit OpenGWAS database at http://app.mrbase.org.

## References

[1] Ferlay J EM, Lam F, Colombet M, Mery L, Pineros M, Znaor A, Soerjomataram I, Bray F. (2018) Global cancer observatory: cancer today. International agency for research on cancer, Lyon. https://gco.iarc.fr/today. Accessed 30 Jan 2019

[2] Wentzensen N, Poole EM, Trabert B et al (2016) Ovarian cancer risk factors by histologic subtype: an analysis from the ovarian cancer cohort consortium. J Clin Oncol 34(24):2888–2898. 10.1200/JCO.2016.66.8178 27325851 10.1200/JCO.2016.66.8178 PMC5012665

[3] Risch HA (1998) Hormonal etiology of epithelial ovarian cancer, with a hypothesis concerning the role of androgens and progesterone. J Natl Cancer Inst 90(23):1774–1786. 10.1093/jnci/90.23.1774 9839517 10.1093/jnci/90.23.1774

[4] Modugno F, Laskey R, Smith AL, Andersen CL, Haluska P, Oesterreich S (2012) Hormone response in ovarian cancer: time to reconsider as a clinical target? Endocr Relat Cancer 19(6):R255–R279. 10.1530/ERC-12-0175 23045324 10.1530/ERC-12-0175 PMC3696394

[5] Lindgren PR, Cajander S, Backstrom T, Gustafsson JA, Makela S, Olofsson JI (2004) Estrogen and progesterone receptors in ovarian epithelial tumors. Mol Cell Endocrinol 221(1–2):97–104. 10.1016/j.mce.2004.02.020 15223136 10.1016/j.mce.2004.02.020

[6] Gapstur SM, Patel AV, Banks E et al (2015) Menopausal hormone use and ovarian cancer risk: individual participant meta-analysis of 52 epidemiological studies. Lancet 385(9980):1835–1842. 10.1016/S0140-6736(14)61687-1 25684585 10.1016/S0140-6736(14)61687-1 PMC4427760

[7] Smith GD, Ebrahim S (2003) “Mendelian randomization”: can genetic epidemiology contribute to understanding environmental determinants of disease? Int J Epidemiol 32(1):1–22. 10.1093/ije/dyg070 12689998 10.1093/ije/dyg070

[8] Phelan CM, Kuchenbaecker KB, Tyrer JP et al (2017) Identification of 12 new susceptibility loci for different histotypes of epithelial ovarian cancer. Nat Genet 49(5):680–691. 10.1038/ng.3826 28346442 10.1038/ng.3826 PMC5612337

[9] Schmitz D, Ek WE, Berggren E, Hoglund J, Karlsson T, Johansson A (2021) Genome-wide association study of estradiol levels, and the causal effect of estradiol on bone mineral density. J Clin Endocrinol Metab. 10.1210/clinem/dgab507 34255042 10.1210/clinem/dgab507 PMC8530739

[10] Prescott J, Thompson DJ, Kraft P et al (2012) Genome-wide association study of circulating estradiol, testosterone, and sex hormone-binding globulin in postmenopausal women. PLoS ONE 7(6):e37815. 10.1371/journal.pone.0037815 22675492 10.1371/journal.pone.0037815 PMC3366971

[11] Tremollieres F, Ribot C (2010) Bone mineral density and prediction of non-osteoporotic disease. Maturitas 65(4):348–351. 10.1016/j.maturitas.2009.12.023 20079983 10.1016/j.maturitas.2009.12.023

[12] Nelson RL, Turyk M, Kim J, Persky V (2002) Bone mineral density and the subsequent risk of cancer in the NHANES I follow-up cohort. BMC Cancer 2(1):22 12377099 10.1186/1471-2407-2-22 PMC130028

[13] Myers TA, Chanock SJ, Machiela MJ (2020) LDlinkR: an R package for rapidly calculating linkage disequilibrium statistics in diverse populations. Front Genet 11:157. 10.3389/fgene.2020.00157 32180801 10.3389/fgene.2020.00157 PMC7059597

[14] Zheng HF, Forgetta V, Hsu YH et al (2015) Whole-genome sequencing identifies EN1 as a determinant of bone density and fracture. Nature 526(7571):112–117. 10.1038/nature14878 26367794 10.1038/nature14878 PMC4755714

[15] Zhu X, Bai W, Zheng H (2021) Twelve years of GWAS discoveries for osteoporosis and related traits: advances, challenges and applications. Bone Res 9(1):23. 10.1038/s41413-021-00143-3 33927194 10.1038/s41413-021-00143-3 PMC8085014

[16] Fujita M, Ogawa S, Fukuoka H et al (2002) Differential expression of secreted frizzled-related protein 4 in decidual cells during pregnancy. J Mol Endocrinol 28(3):213–223. 10.1677/jme.0.0280213 12063187 10.1677/jme.0.0280213

[17] Sikora MJ, Jacobsen BM, Levine K et al (2016) WNT4 mediates estrogen receptor signaling and endocrine resistance in invasive lobular carcinoma cell lines. Breast Cancer Res 18(1):92. 10.1186/s13058-016-0748-7 27650553 10.1186/s13058-016-0748-7 PMC5028957

[18] Soares R, Balogh G, Guo S, Gartner F, Russo J, Schmitt F (2004) Evidence for the notch signaling pathway on the role of estrogen in angiogenesis. Mol Endocrinol 18(9):2333–2343. 10.1210/me.2003-0362 15192074 10.1210/me.2003-0362

[19] Veeraraghavan J, Tan Y, Cao XX et al (2014) Recurrent ESR1-CCDC170 rearrangements in an aggressive subset of oestrogen receptor-positive breast cancers. Nat Commun 5:4577. 10.1038/ncomms5577 25099679 10.1038/ncomms5577 PMC4130357

[20] Gao Y, Huang E, Zhang H et al (2013) Crosstalk between Wnt/beta-catenin and estrogen receptor signaling synergistically promotes osteogenic differentiation of mesenchymal progenitor cells. PLoS ONE 8(12):e82436. 10.1371/journal.pone.0082436 24340027 10.1371/journal.pone.0082436 PMC3855436

[21] Newcomb PA, Trentham-Dietz A, Egan KM et al (2001) Fracture history and risk of breast and endometrial cancer. Am J Epidemiol 153(11):1071–1078. 10.1093/aje/153.11.1071 11390325 10.1093/aje/153.11.1071

[22] Qu X, Zhang X, Qin A et al (2013) Bone mineral density and risk of breast cancer in postmenopausal women. Breast Cancer Res Treat 138(1):261–271. 10.1007/s10549-013-2431-3 23381744 10.1007/s10549-013-2431-3

[23] Buist DS, LaCroix AZ, Barlow WE, White E, Weiss NS (2001) Bone mineral density and breast cancer risk in postmenopausal women. J Clin Epidemiol 54(4):417–422. 10.1016/s0895-4356(00)00301-2 11297892 10.1016/s0895-4356(00)00301-2

[24] Danforth KN, Schairer C, Schatzkin A, Lacey JV (2009) Bone fractures and incident epithelial ovarian cancer in a prospective cohort study. J Womens Health 18(11):1777–1782. 10.1089/jwh.2008.1341 10.1089/jwh.2008.1341 PMC2828254 19951211

[25] Bowden J, Davey Smith G, Haycock PC, Burgess S (2016) Consistent estimation in mendelian randomization with some invalid instruments using a weighted median estimator. Genet Epidemiol 40(4):304–314. 10.1002/gepi.21965 27061298 10.1002/gepi.21965 PMC4849733

[26] Martens EP, Pestman WR, de Boer A, Belitser SV, Klungel OH (2006) Instrumental variables: application and limitations. Epidemiology 17(3):260–267. 10.1097/01.ede.0000215160.88317.cb 16617274 10.1097/01.ede.0000215160.88317.cb

[27] Grant AJ, Burgess S (2021) Pleiotropy robust methods for multivariable Mendelian randomization. Stat Med. 10.1002/sim.9156 34342032 10.1002/sim.9156 PMC7612169

[28] Lane NE (2006) Epidemiology, etiology, and diagnosis of osteoporosis. Am J Obstet Gynecol 194(2 Suppl):S3-11. 10.1016/j.ajog.2005.08.047 16448873 10.1016/j.ajog.2005.08.047

[29] Whiteman DC, Webb PM, Green AC et al (2015) Cancers in Australia in 2010 attributable to modifiable factors: summary and conclusions. Aust N Z J Public Health 39(5):477–484. 10.1111/1753-6405.12471 26437735 10.1111/1753-6405.12471 PMC4606779

[30] Hannan MT, Felson DT, Dawson-Hughes B, Tucker KL, Cupples LA, Wilson PW, Kiel DP (2000) Risk factors for longitudinal bone loss in elderly men and women: the Framingham osteoporosis study. J Bone Miner Res 15(4):710–720. 10.1359/jbmr.2000.15.4.710 10780863 10.1359/jbmr.2000.15.4.710

[31] Hartley A, Sanderson E, Granell R et al (2022) Using multivariable Mendelian randomization to estimate the causal effect of bone mineral density on osteoarthritis risk, independently of body mass index. Int J Epidemiol 51(4):1254–1267. 10.1093/ije/dyab251 34897459 10.1093/ije/dyab251 PMC9365636

[32] Placeholder for repository for Supplementary File. [database on the Internet].

[33] Hemani G, Zheng J, Elsworth B et al (2018) The MR-Base platform supports systematic causal inference across the human phenome. Elife. 10.7554/eLife.34408 29846171 10.7554/eLife.34408 PMC5976434

[34] Mitchell RC, Elsworth, Bl, et al., editors. MRC IEU UK Biobank GWAS pipeline version 22019.

[35] Abbott LB, S Churchhouse C, Ganna A, Howrigan H, Palmer D, Neale B, Walters R, Carey C The Hail team. Neale lab UKB Round 2 GWAS summary statistics. Neale lab. 2018. http://www.nealelab.is/uk-biobank/. 2022.

[36] Hemani G, Tilling K, Davey SG (2017) Orienting the causal relationship between imprecisely measured traits using GWAS summary data. PLoS Genet 13(11):e1007081. 10.1371/journal.pgen.1007081 29149188 10.1371/journal.pgen.1007081 PMC5711033

[37] R Core Team (2021) R: A language and environment for statistical computing. R Foundation for Statistical Computing, Vienna

[38] Chen Z, Boehnke M, Wen X, Mukherjee B (2021) Revisiting the genome-wide significance threshold for common variant GWAS. Genes Genom Genet. 10.1093/g3journal/jkaa056 10.1093/g3journal/jkaa056 PMC8022962 33585870

[39] Zhang H, Ma K, Li R-M, Li J-N, Gao S-F, Ma L-N (2022) Association between testosterone levels and bone mineral density in females aged 40–60 years from NHANES 2011–2016. Sci Rep 12(1):16426. 10.1038/s41598-022-21008-7 36180560 10.1038/s41598-022-21008-7 PMC9525583

[40] Burgess S, Thompson SG, Collaboration CCG (2011) Avoiding bias from weak instruments in Mendelian randomization studies. Int J Epidemiol 40(3):755–764. 10.1093/ije/dyr036 21414999 10.1093/ije/dyr036

[41] Burgess S, Thompson SG (2017) Interpreting findings from Mendelian randomization using the MR-Egger method. Eur J Epidemiol 32(5):377–389. 10.1007/s10654-017-0255-x 28527048 10.1007/s10654-017-0255-x PMC5506233

[42] Zhu X, Li X, Xu R, Wang T (2021) An iterative approach to detect pleiotropy and perform Mendelian randomization analysis using GWAS summary statistics. Bioinformatics 37(10):1390–1400. 10.1093/bioinformatics/btaa985 33226062 10.1093/bioinformatics/btaa985 PMC8208738

[43] Zhao Q, Wang J, Gibran H, Bowden J, Small DS (2019) Statistical inference in two-sample summary-data Mendelian randomization using robust adjusted profile score. arXiv. 10.48550/arxiv.1801.09652

[44] Qi G, Chatterjee N (2019) Mendelian randomization analysis using mixture models for robust and efficient estimation of causal effects. Nat Commun 10(1):1941. 10.1038/s41467-019-09432-2 31028273 10.1038/s41467-019-09432-2 PMC6486646

[45] RStudio Team (2021) RStudio: Integrated Development for R RStudio. PBC, Boston

[46] Burgess S, Davey Smith G, Davies NM et al (2019) Guidelines for performing Mendelian randomization investigations. Wellcome Open Res 4:186. 10.12688/wellcomeopenres.15555.2 32760811 10.12688/wellcomeopenres.15555.1 PMC7384151

[47] Skrivankova VW, Richmond RC, Woolf BAR et al (2021) Strengthening the reporting of observational studies in epidemiology using Mendelian randomization: the strobe-MR statement. JAMA 326(16):1614–1621. 10.1001/jama.2021.18236 34698778 10.1001/jama.2021.18236

[48] Gharwan H, Bunch KP, Annunziata CM (2015) The role of reproductive hormones in epithelial ovarian carcinogenesis. Endocr Relat Cancer 22(6):R339–R363. 10.1530/ERC-14-0550 26373571 10.1530/ERC-14-0550

[49] Estrada K, Styrkarsdottir U, Evangelou E et al (2012) Genome-wide meta-analysis identifies 56 bone mineral density loci and reveals 14 loci associated with risk of fracture. Nat Genet 44(5):491–501. 10.1038/ng.2249 22504420 10.1038/ng.2249 PMC3338864

[50] Rivadeneira F, Styrkarsdottir U, Estrada K et al (2009) Twenty bone-mineral-density loci identified by large-scale meta-analysis of genome-wide association studies. Nat Genet 41(11):1199–1206. 10.1038/ng.446 19801982 10.1038/ng.446 PMC2783489

[51] Elsworth B, Lyon M, Alexander T et al (2020) The MRC IEU OpenGWAS data infrastructure. bioRxiv. 10.1101/2020.08.10.244293

