# Supplementary material for: Exploring estrogen-related mechanisms in ovarian carcinogenesis: association between bone mineral density and ovarian cancer risk in a multivariable Mendelian randomization study
Source: Cancer Causes Control. 2024 Oct 18;36(2):171–82. doi: 10.1007/s10552-024-01926-9 (PMC11775049; doi:10.1007/s10552-024-01926-9)
Supplement: Supplementary file 1 — Supplementary file1 (PDF 817 KB) [file 10552_2024_1926_MOESM1_ESM.pdf]

Supplementary Materials

This document contains supplementary materials for the following manuscript:

Journal: Cancer Causes & Control

Title: Exploring oestrogen-related mechanisms in ovarian carcinogenesis: Association between bone mineral density and ovarian cancer risk in a multivariable Mendelian randomisation study

Authors: Karen M. Tuesley, Penelope M. Webb, Melinda M. Protani, Peter Donovan, Susan J. Jordan, Suzanne Dixon-Suen

Corresponding Author: Dr Karen M. Tuesley, School of Public Health, The University of Queensland, Brisbane, Queensland, Australia, k.tuesley@uq.edu.au

Table of Contents

List of Supplementary Tables 1

List of Supplementary Figures 1

Supplementary Methods 3

References 4

Supplementary Tables 5

Supplementary Figures 7

List of Supplementary Tables

Supplementary Table 1: Linkage disequilibrium for SNPs associated with oestradiol in females and bone mineral density 5

Supplementary Table 2: Invalid SNPs removed for MVMR-Lasso analysis 5

Supplementary Table 3: Multivariable Mendelian randomisation analyses for the association between lumbar spine and femoral neck bone mineral density and risk of high grade serous and endometrioid epithelial ovarian cancers, separately adjusting for each covariate and including testosterone 6

List of Supplementary Figures

Supplementary Figure 1: Univariate Mendelian randomisation analyses for lumbar spine bone mineral density as the exposure and high grade serous epithelial ovarian cancer as the outcome 7

Supplementary Figure 2: Univariate Mendelian randomisation analyses for femoral neck bone mineral density as the exposure and high grade serous epithelial ovarian cancer as the outcome 8

Supplementary Figure 3: Univariate Mendelian randomisation analyses for lumbar spine bone mineral density as the exposure and low grade serous epithelial ovarian cancer as the outcome 9

Supplementary Figure 4: Univariate Mendelian randomisation analyses for femoral neck bone mineral density as the exposure and low grade serous epithelial ovarian cancer as the outcome..... 10
Supplementary Figure 5: Univariate Mendelian randomisation analyses for lumbar spine bone mineral density as the exposure and endometrioid epithelial ovarian cancer as the outcome. 11
Supplementary Figure 6: Univariate Mendelian randomisation analyses for femoral neck bone mineral density as the exposure and endometrioid epithelial ovarian cancer as the outcome. 12
Supplementary Figure 7: Univariate Mendelian randomisation analyses for lumbar spine bone mineral density as the exposure and clear cell epithelial ovarian cancer as the outcome. 13
Supplementary Figure 8: Univariate Mendelian randomisation analyses for femoral neck bone mineral density as the exposure and clear cell epithelial ovarian cancer as the outcome. 14
Supplementary Figure 9: Univariate Mendelian randomisation analyses for lumbar spine bone mineral density as the exposure and invasive mucinous epithelial ovarian cancer as the outcome... 15
Supplementary Figure 10: Univariate Mendelian randomisation analyses for femoral neck bone mineral density as the exposure and invasive mucinous epithelial ovarian cancer as the outcome... 16

Supplementary Methods

Oestradiol-associated SNPs and bone mineral density

We reviewed linkage disequilibrium (LD)[1] between two single nucleotide polymorphisms (SNPs) associated ($p < 5*10^{-8}$) with oestradiol levels in females[2] and the SNPs associated with femoral neck and lumbar spine bone mineral density (BMD). Supplementary Table 1 shows the results. Both SNPs have moderate to high correlation with at least one SNP associated with BMD; with lumbar spine BMD having high correlation with oestradiol SNP rs45446698.

References

1. Myers TA, Chanock SJ, Machiela MJ. LDlinkR: An R Package for Rapidly Calculating Linkage Disequilibrium Statistics in Diverse Populations. Front Genet. 2020;11:157

2. Schmitz D, Ek WE, Berggren E, Hoglund J, Karlsson T, Johansson A. Genome-Wide Association Study of Estradiol Levels, and the Causal Effect of Estradiol on Bone Mineral Density. J Clin Endocrinol Metab. 2021

Supplementary Tables

Supplementary Table 1: Linkage disequilibrium for SNPs associated with oestradiol in females and bone mineral density

Oestradiol SNP	location	BMD SNP	location	type	Linkage disequilibrium
rs16991615	chr20:5948227	rs2235811	chr20:10644158	lumbar spine	0.305
rs45446698	chr7:99332948	rs35681117	chr7:37959021	lumbar spine	1.000
		rs1357651	chr7:38097862	lumbar spine	0.484
		rs3779381	chr7:120966790	femoral neck	0.233

BMD: bone mineral density; SNP: single nucleotide polymorphisms.

Supplementary Table 2: Invalid SNPs removed for MVMR-Lasso analysis

BMD exposure	EOC outcome	Variants	Valid SNPs	Invalid SNPs BMD	Invalid SNPs confounding variables
Lumbar spine	High-grade serous	1476	1431	0	45
	Low-grade serous	1478	1476	0	2
	Endometrioid	1478	1452	0	26
	Clear cell	1478	1433	1	44
	Invasive mucinous	1478	1460	0	18
Femoral neck	High-grade serous	1475	1432	0	43
	Low-grade serous	1477	1475	0	2
	Endometrioid	1477	1459	0	18
	Clear cell	1477	1434	0	43
	Invasive mucinous	1477	1459	0	18

BMD: bone mineral density; EOC: epithelial ovarian cancer; MVMR: multivariable Mendelian randomisation; SNP: single nucleotide polymorphisms.

Supplementary Table 3: Multivariable Mendelian randomisation analyses for the association between lumbar spine and femoral neck bone mineral density and risk of high grade serous and endometrioid epithelial ovarian cancers, separately adjusting for each covariate and including testosterone.

Method	High-grade serous OR (95% CI)	Endometrioid OR (95% CI)
**Lumbar spine BMD**		
Univariable MR-IVW	1.16 (1.03, 1.30)	1.21 (0.93, 1.57)
Multivariable MR-IVW		
Adjusted for BMI	1.09 (0.99, 1.21)	1.11 (0.93, 1.33)
Adjusted for height	1.11 (1.02, 1.21)	1.14 (0.98, 1.33)
Adjusted for smoking	1.16 (1.03, 1.30)	1.21 (0.95, 1.54)
Adjusted for alcohol	1.09 (0.96, 1.24)	1.13 (0.90, 1.41)
Adjusted for age at menarche	1.10 (0.99, 1.22)	1.25 (1.02, 1.54)
Adjusted for age at menopause	1.20 (1.07, 1.35)	1.28 (1.06, 1.56)
Adjusted for vitamin D	1.14 (1.02, 1.29)	1.18 (0.95, 1.47)
Adjusted for testosterone	1.14 (1.02, 1.28)	1.22 (1.00, 1.50)
Adjusted for all covariates excluding testosterone	1.06 (0.99, 1.14)	1.13 (1.00, 1.28)
Adjusted for all covariates including testosterone	1.06 (0.99, 1.13)[a]	1.14 (1.01, 1.28)[a]
**Femoral neck BMD**		
Univariable MR-IVW	1.12 (0.97, 1.29)	1.25 (0.99, 1.57)
Multivariable MR-IVW		
Adjusted for BMI	1.09 (0.98, 1.22)	1.15 (0.95, 1.39)
Adjusted for height	1.04 (0.94, 1.14)	1.21 (1.03, 1.43)
Adjusted for smoking	1.13 (0.99, 1.28)	1.28 (1.02, 1.62)
Adjusted for alcohol	1.10 (0.96, 1.27)	1.24 (0.98, 1.57)
Adjusted for age at menarche	1.08 (0.95, 1.23)	1.18 (0.93, 1.48)
Adjusted for age at menopause	1.16 (1.01, 1.33)	1.35 (1.08, 1.68)
Adjusted for vitamin D	1.08 (0.94, 1.24)	1.21 (0.97, 1.52)
Adjusted for testosterone	1.13 (1.00, 1.28)	1.17 (0.94, 1.46)
Adjusted for all covariates excluding testosterone	1.03 (0.96, 1.12)	1.19 (1.03, 1.36)
Adjusted for all covariates including testosterone	1.04 (0.97, 1.13)[a]	1.16 (1.02, 1.33)[a]

BMD: bone mineral density; CI: confidence interval; IVW: inverse-variance weighted; MR: Mendelian Randomisation; MVMR: multivariable Mendelian randomisation; OR: odds ratio.

[a] 20 SNPs from the main analysis were excluded in this sensitivity analysis. 3 SNPs were excluded, one for each of BMI, height and age at menopause, as they were also associated with testosterone. 17 SNPs were excluded as there was no association available for testosterone.

Supplementary Figures

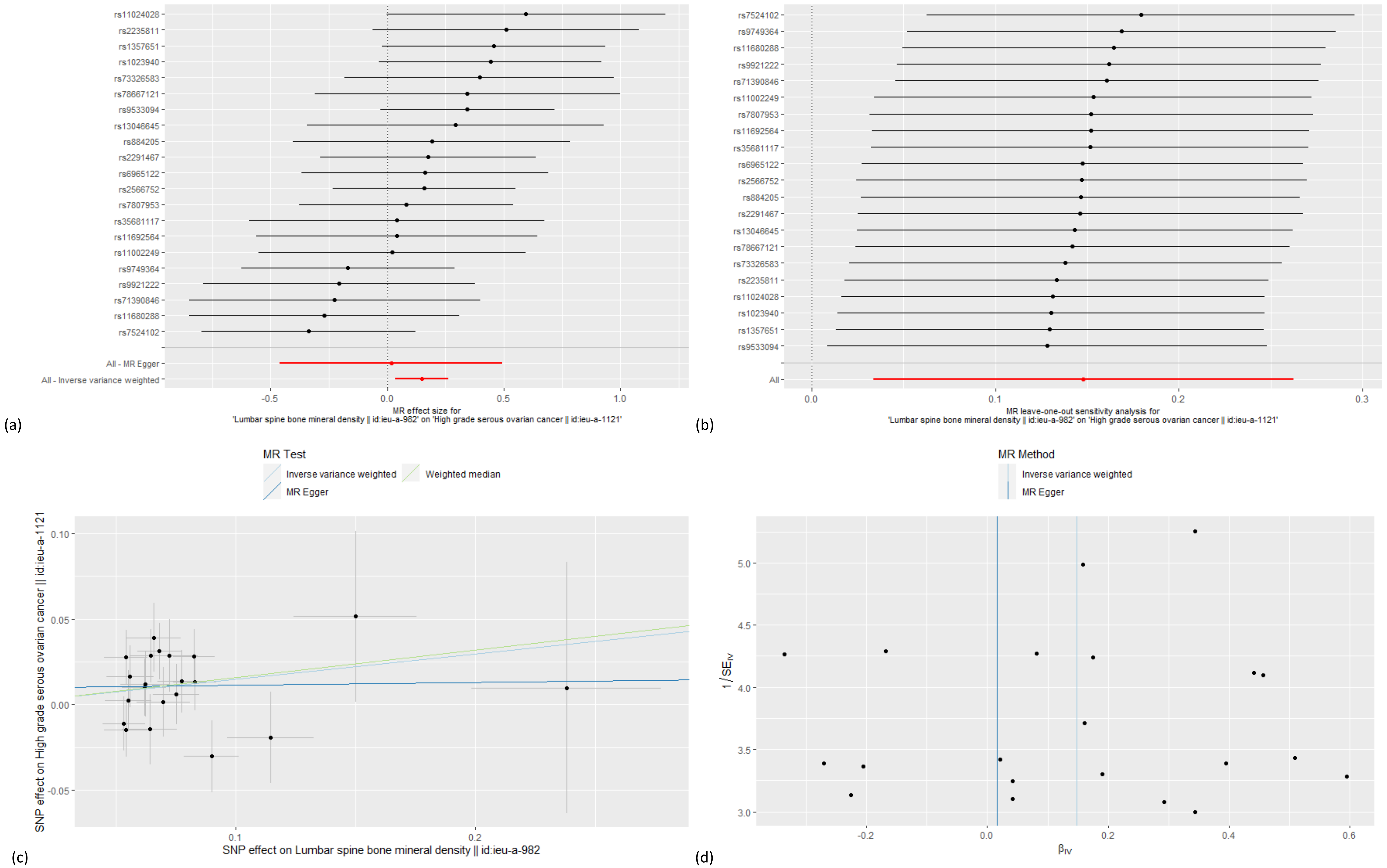

<u>Supplementary Figure 1: Univariate Mendelian randomisation analyses for lumbar spine bone mineral density as the exposure and high grade serous epithelial ovarian cancer as the outcome.</u>
(a) Forest plot showing the effect size for each SNP and for all SNPs combined. (b) Forest plot showing the overall MR estimate leaving out one SNP at a time (All = All inverse variance weighted). (c) Scatter plot showing the association of each SNP with lumbar spine BMD vs. the association with high grade serous EOC. (d) Funnel plot of instrument precision against the MR estimate for each variant.
BMD: bone mineral density; EOC: epithelial ovarian cancer; MR: Mendelian randomisation; SNP: single nucleotide polymorphisms.

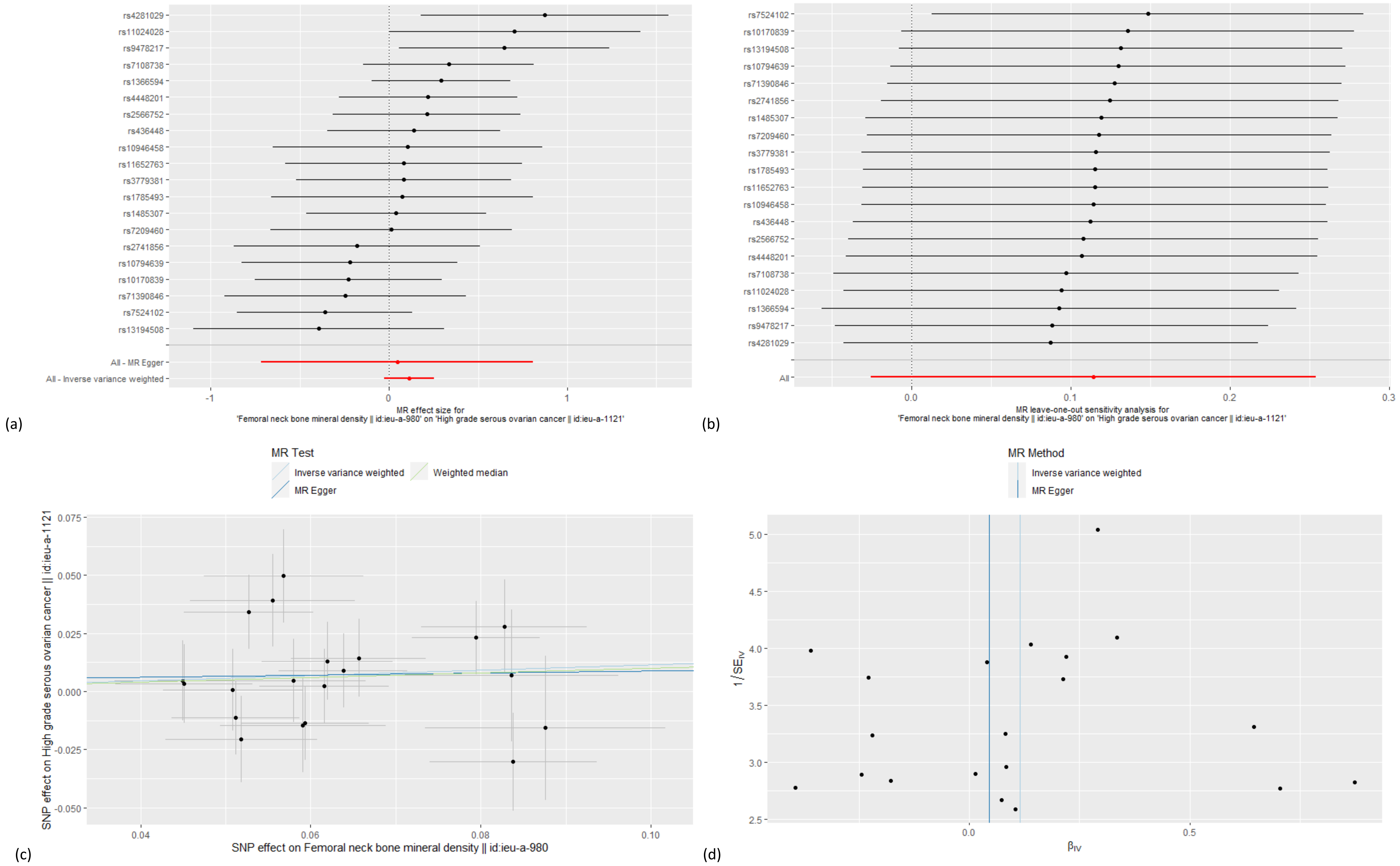

<u>Supplementary Figure 2: Univariate Mendelian randomisation analyses for femoral neck bone mineral density as the exposure and high grade serous epithelial ovarian cancer as the outcome.</u>
(a) Forest plot showing the effect size for each SNP and for all SNPs combined. (b) Forest plot showing the overall MR estimate leaving out one SNP at a time (All = All inverse variance weighted). (c) Scatter plot showing the association of each SNP with femoral neck BMD vs. the association with high grade serous EOC. (d) Funnel plot of instrument precision against the MR estimate for each variant.
BMD: bone mineral density; EOC: epithelial ovarian cancer; MR: Mendelian randomisation; SNP: single nucleotide polymorphisms.

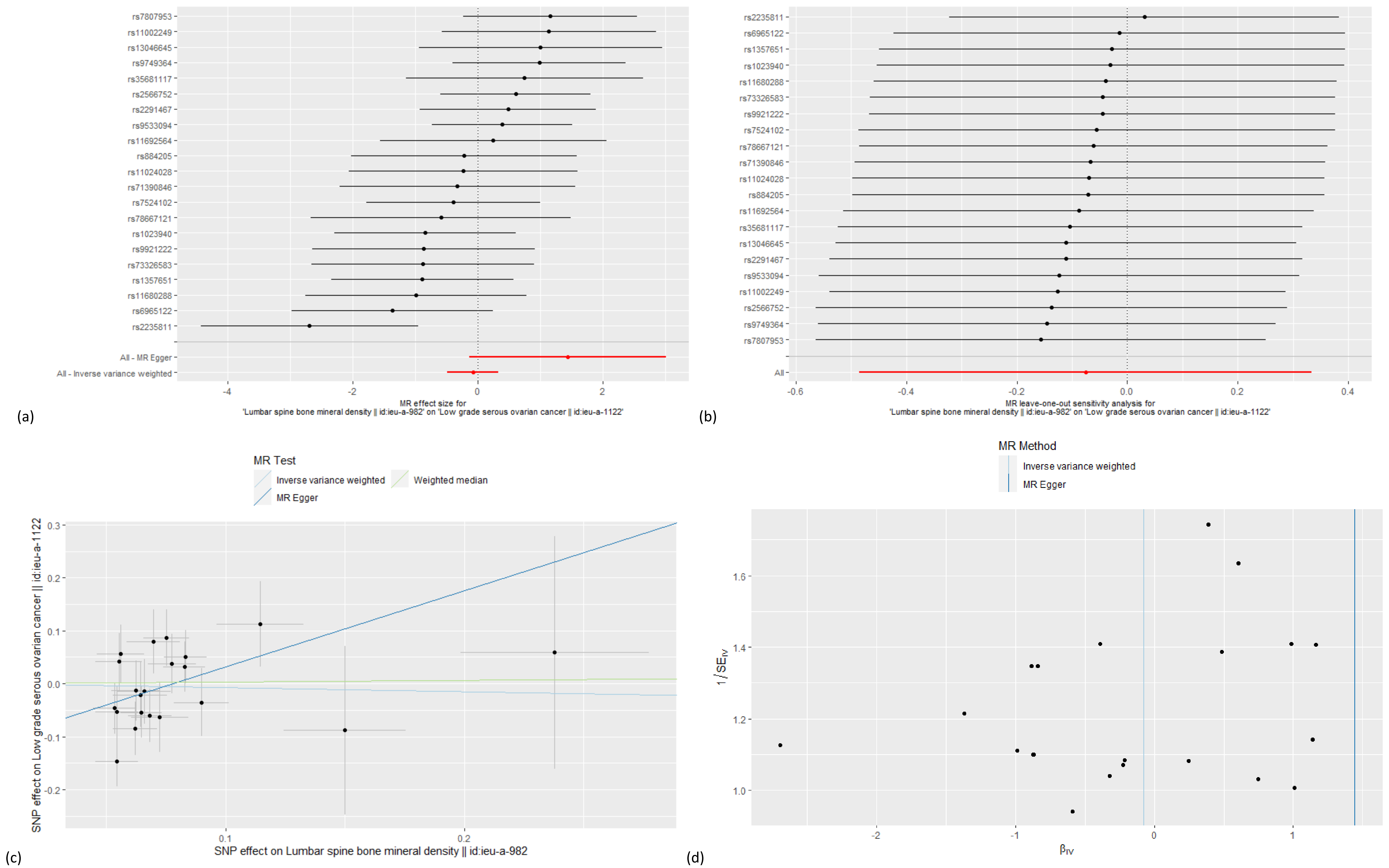

Supplementary Figure 3: Univariate Mendelian randomisation analyses for lumbar spine bone mineral density as the exposure and low grade serous epithelial ovarian cancer as the outcome.

(a) Forest plot showing the effect size for each SNP and for all SNPs combined. (b) Forest plot showing the overall MR estimate leaving out one SNP at a time (All = All inverse variance weighted). (c) Scatter plot showing the association of each SNP with lumbar spine BMD vs. the association with low grade serous EOC. (d) Funnel plot of instrument precision against the MR estimate for each variant.

BMD: bone mineral density; EOC: epithelial ovarian cancer; MR: Mendelian randomisation; SNP: single nucleotide polymorphisms.

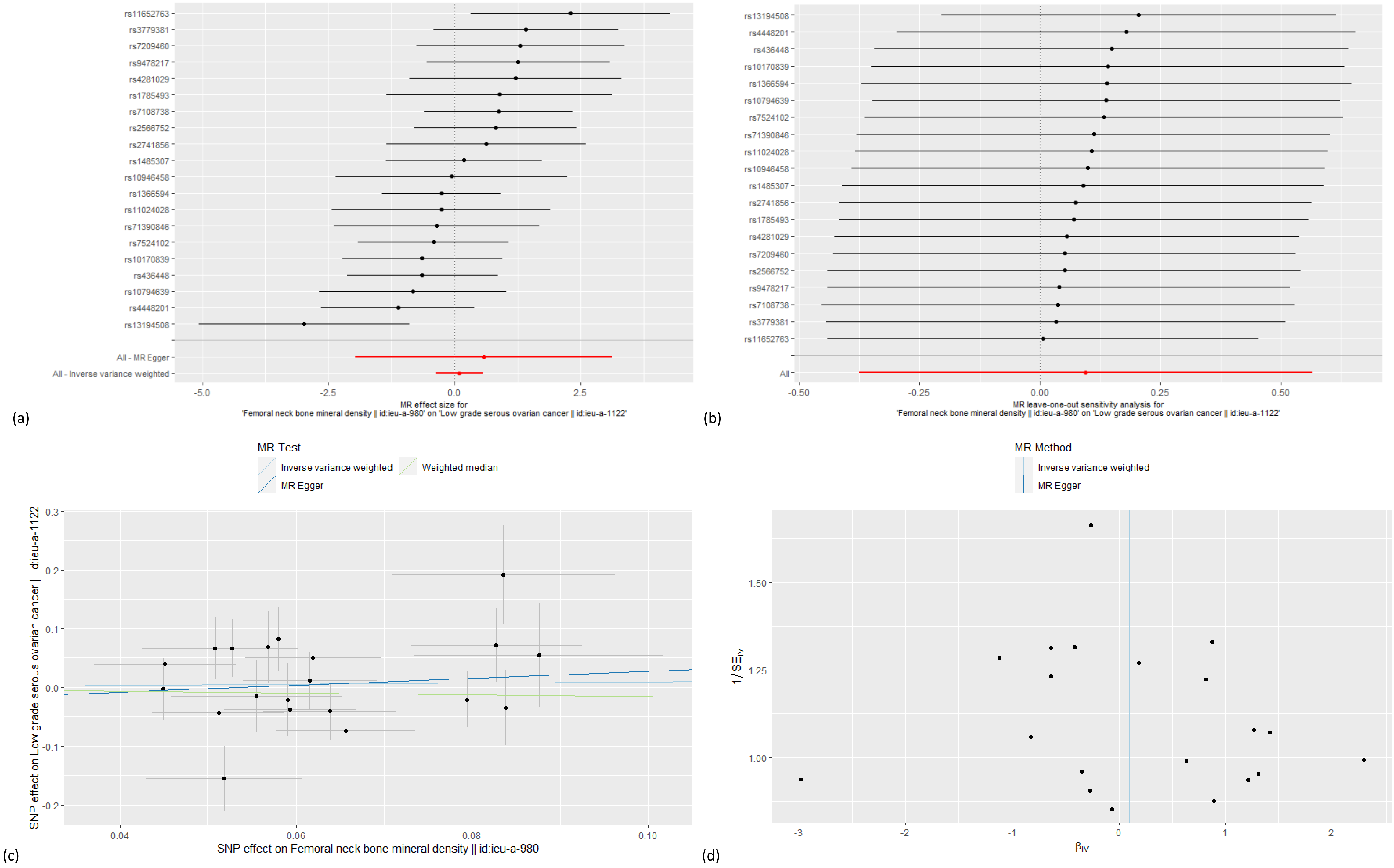

Supplementary Figure 4: Univariate Mendelian randomisation analyses for femoral neck bone mineral density as the exposure and low grade serous epithelial ovarian cancer as the outcome.

(a) Forest plot showing the effect size for each SNP and for all SNPs combined. (b) Forest plot showing the overall MR estimate leaving out one SNP at a time (All = All inverse variance weighted). (c) Scatter plot showing the association of each SNP with femoral neck BMD vs. the association with low grade serous EOC. (d) Funnel plot of instrument precision against the MR estimate for each variant.

BMD: bone mineral density; EOC: epithelial ovarian cancer; MR: Mendelian randomisation; SNP: single nucleotide polymorphisms.

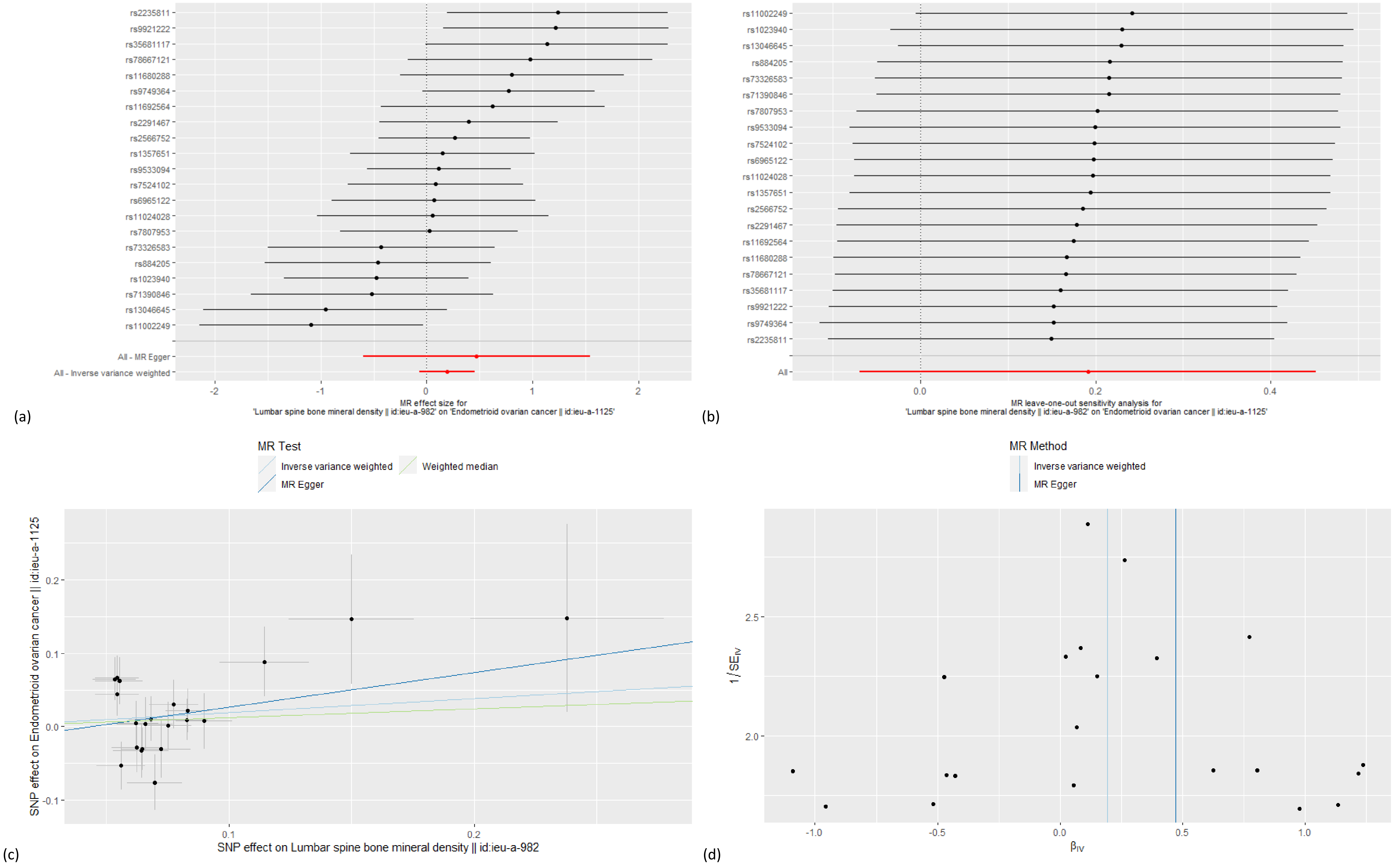

<u>Supplementary Figure 5: Univariate Mendelian randomisation analyses for lumbar spine bone mineral density as the exposure and endometrioid epithelial ovarian cancer as the outcome.</u>
(a) Forest plot showing the effect size for each SNP and for all SNPs combined. (b) Forest plot showing the overall MR estimate leaving out one SNP at a time (All = All inverse variance weighted). (c) Scatter plot showing the association of each SNP with lumbar spine BMD vs. the association with endometrioid EOC. (d) Funnel plot of instrument precision against the MR estimate for each variant.
BMD: bone mineral density; EOC: epithelial ovarian cancer; MR: Mendelian randomisation; SNP: single nucleotide polymorphisms.

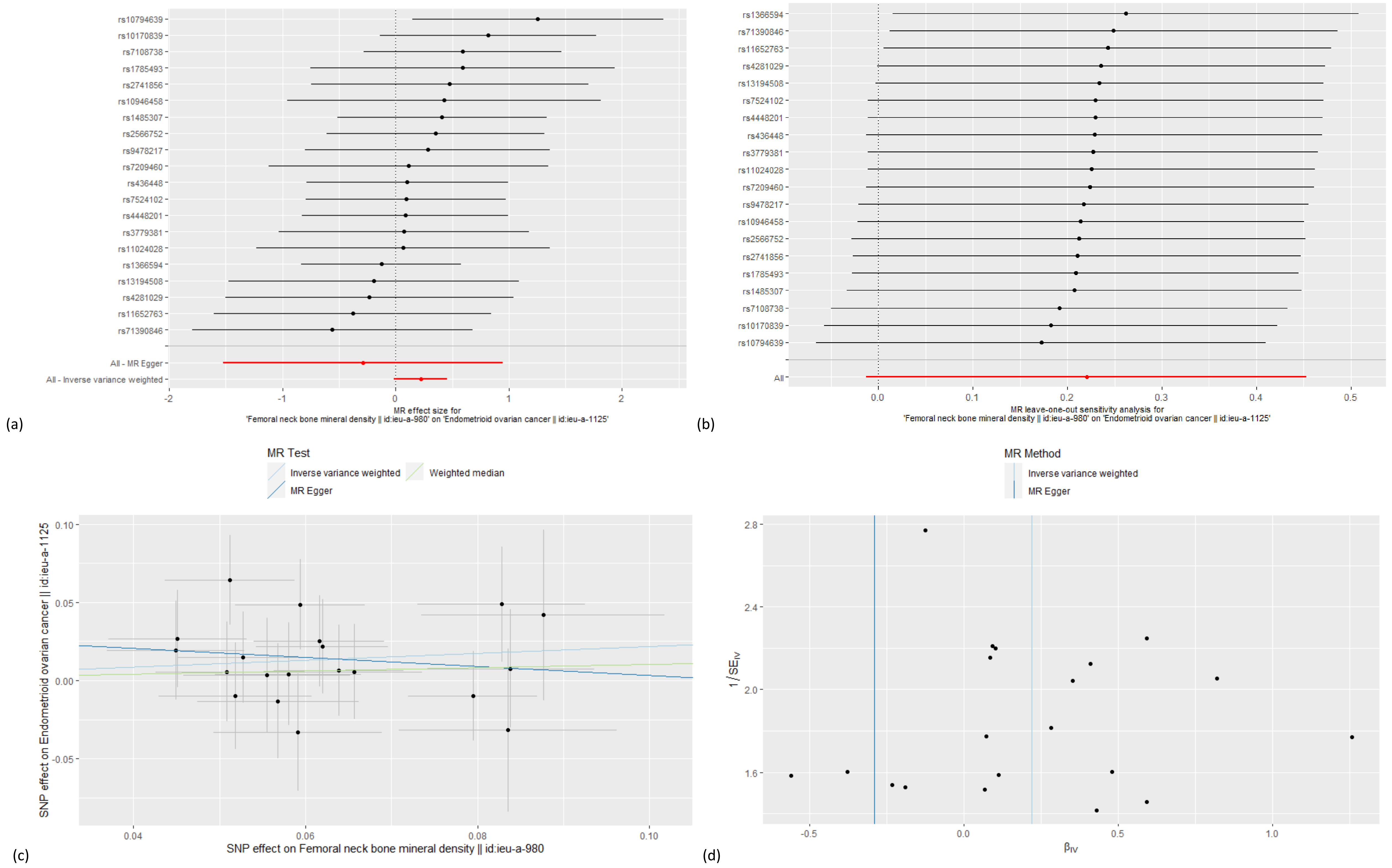

Supplementary Figure 6: Univariate Mendelian randomisation analyses for femoral neck bone mineral density as the exposure and endometrioid epithelial ovarian cancer as the outcome.
(a) Forest plot showing the effect size for each SNP and for all SNPs combined. (b) Forest plot showing the overall MR estimate leaving out one SNP at a time (All = All inverse variance weighted). (c) Scatter plot showing the association of each SNP with femoral neck BMD vs. the association with endometrioid EOC. (d) Funnel plot of instrument precision against the MR estimate for each variant.
BMD: bone mineral density; EOC: epithelial ovarian cancer; MR: Mendelian randomisation; SNP: single nucleotide polymorphisms.

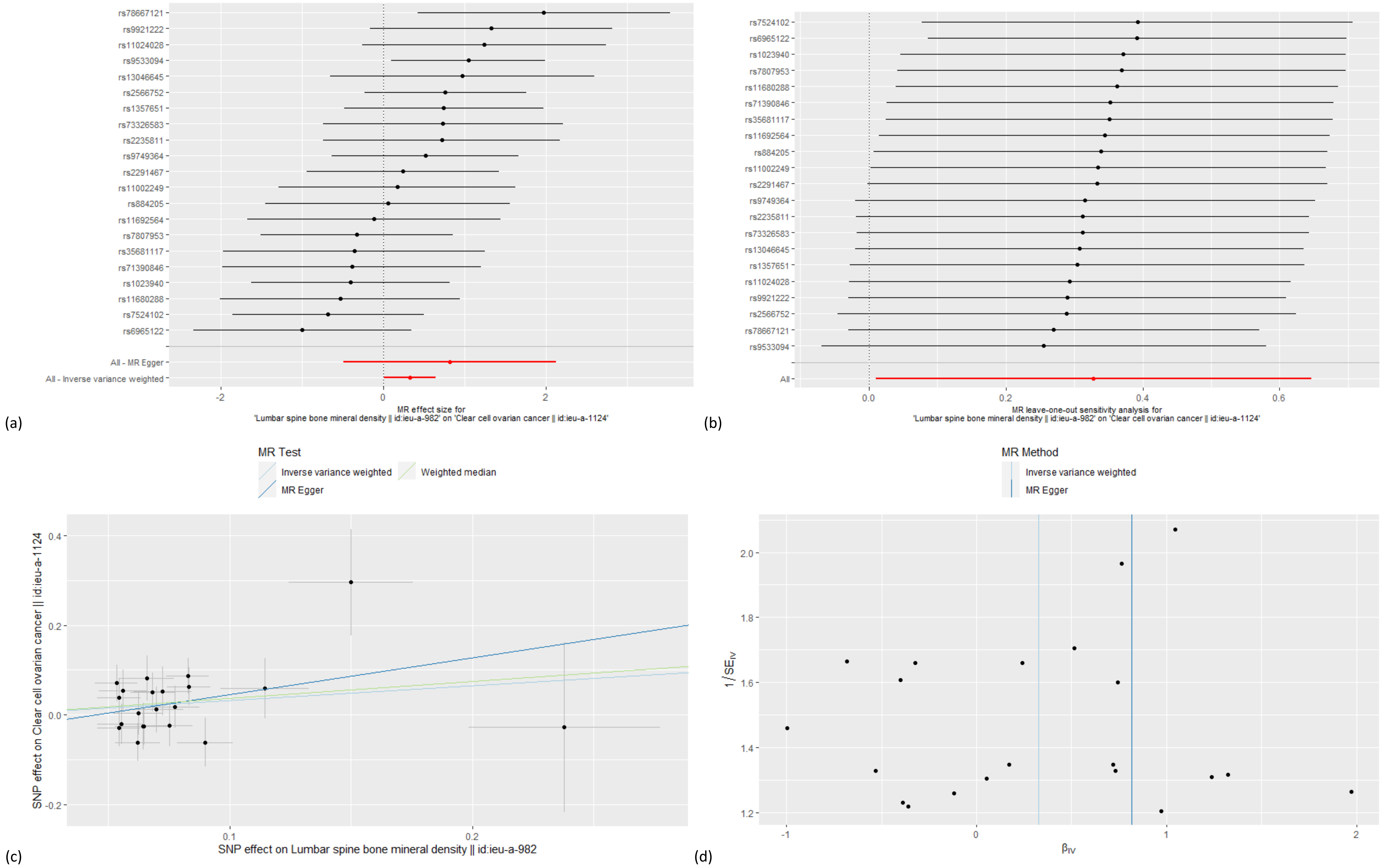

<u>Supplementary Figure 7: Univariate Mendelian randomisation analyses for lumbar spine bone mineral density as the exposure and clear cell epithelial ovarian cancer as the outcome.</u>

(a) Forest plot showing the effect size for each SNP and for all SNPs combined. (b) Forest plot showing the overall MR estimate leaving out one SNP at a time (All = All inverse variance weighted). (c) Scatter plot showing the association of each SNP with lumbar spine BMD vs. the association with clear cell EOC. (d) Funnel plot of instrument precision against the MR estimate for each variant.

BMD: bone mineral density; EOC: epithelial ovarian cancer; MR: Mendelian randomisation; SNP: single nucleotide polymorphisms.

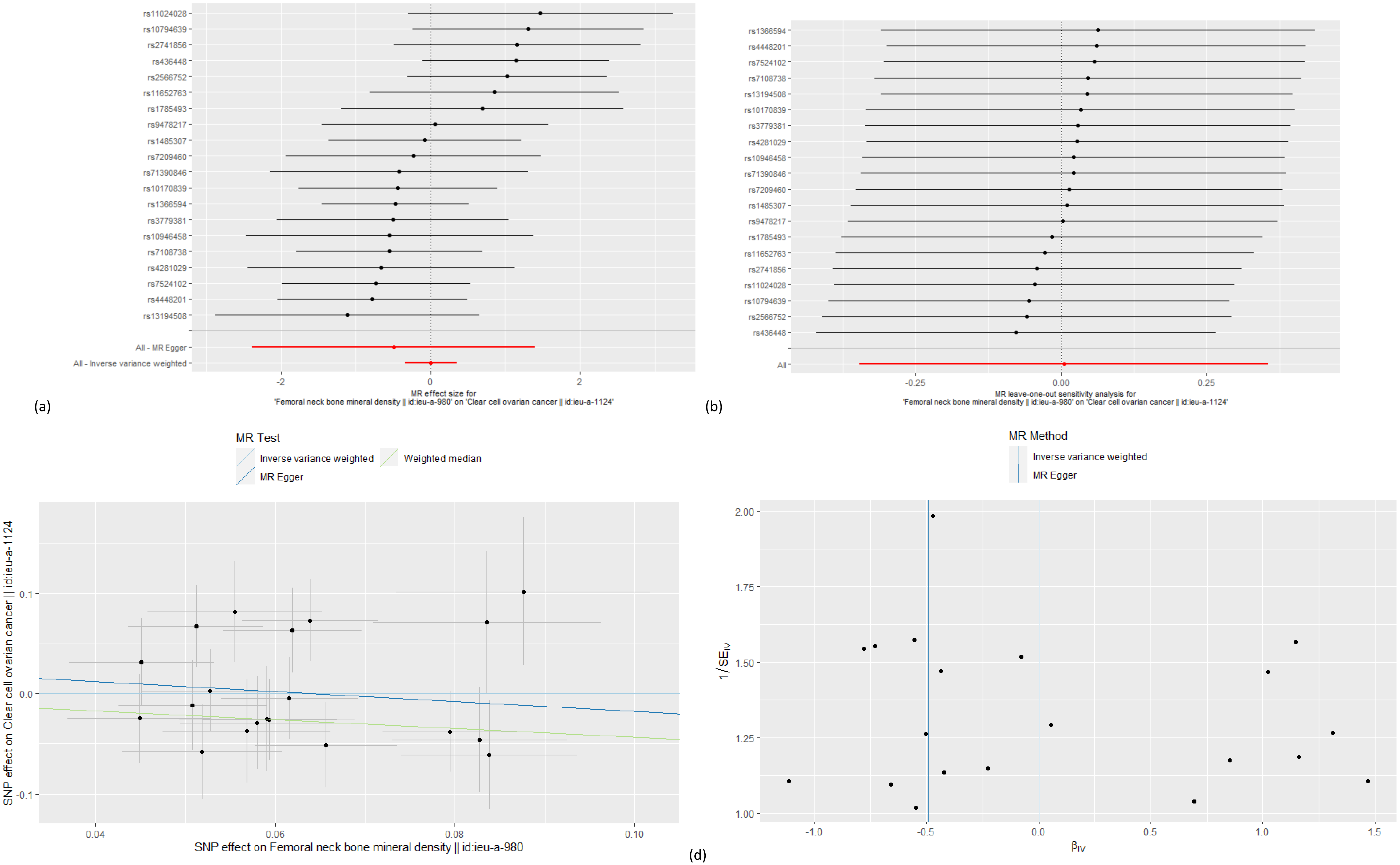

Supplementary Figure 8: Univariate Mendelian randomisation analyses for femoral neck bone mineral density as the exposure and clear cell epithelial ovarian cancer as the outcome.

(a) Forest plot showing the effect size for each SNP and for all SNPs combined. (b) Forest plot showing the overall MR estimate leaving out one SNP at a time (All = All inverse variance weighted). (c) Scatter plot showing the association of each SNP with femoral neck BMD vs. the association with clear cell EOC. (d) Funnel plot of instrument precision against the MR estimate for each variant.

BMD: bone mineral density; EOC: epithelial ovarian cancer; MR: Mendelian randomisation; SNP: single nucleotide polymorphisms.

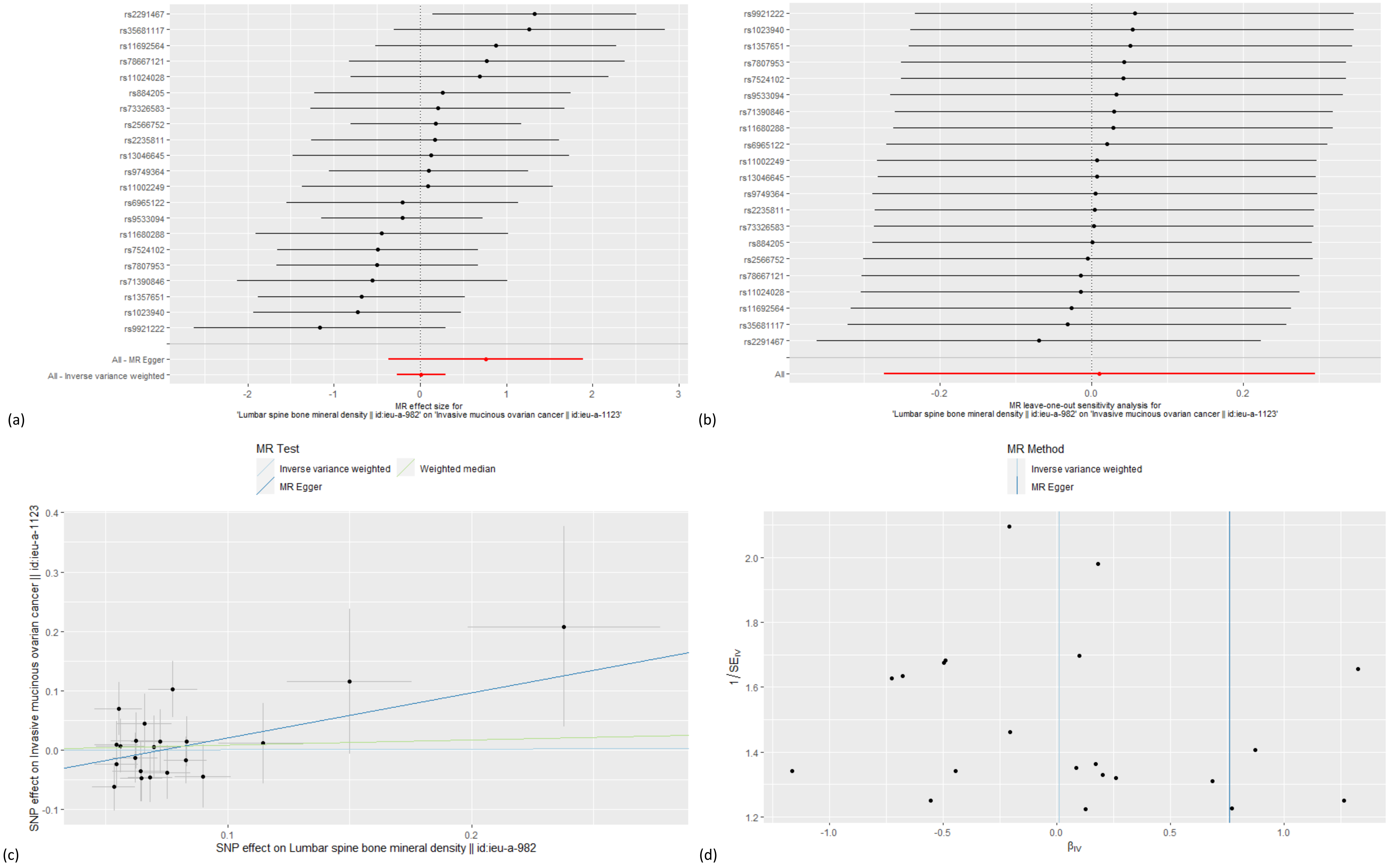

<u>Supplementary Figure 9: Univariate Mendelian randomisation analyses for lumbar spine bone mineral density as the exposure and invasive mucinous epithelial ovarian cancer as the outcome.</u>
(a) Forest plot showing the effect size for each SNP and for all SNPs combined. (b) Forest plot showing the overall MR estimate leaving out one SNP at a time (All = All inverse variance weighted). (c) Scatter plot showing the association of each SNP with lumbar spine BMD vs. the association with invasive mucinous EOC. (d) Funnel plot of instrument precision against the MR estimate for each variant.
BMD: bone mineral density; EOC: epithelial ovarian cancer; MR: Mendelian randomisation; SNP: single nucleotide polymorphisms.

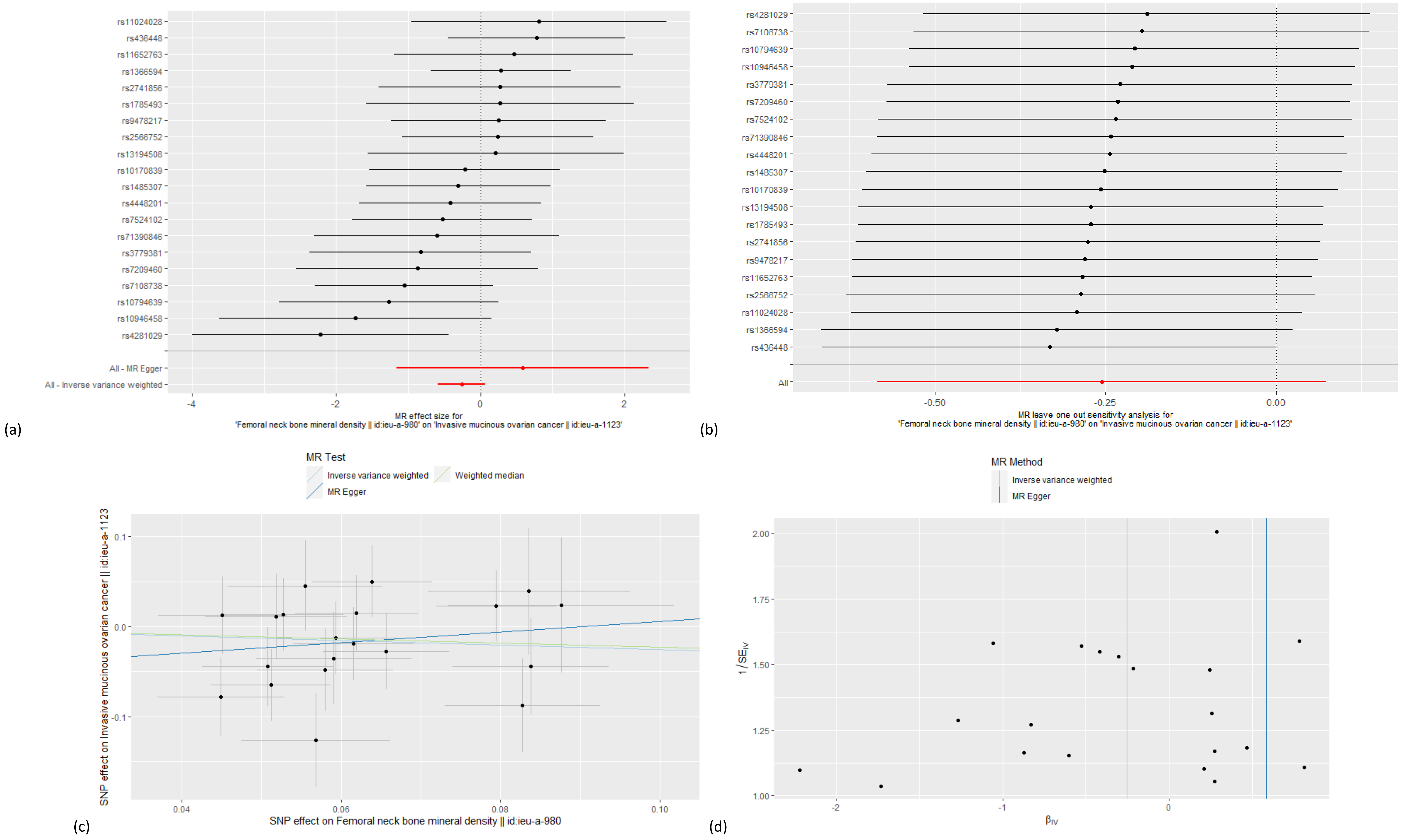

<u>Supplementary Figure 10: Univariate Mendelian randomisation analyses for femoral neck bone mineral density as the exposure and invasive mucinous epithelial ovarian cancer as the outcome.</u>

(a) Forest plot showing the effect size for each SNP and for all SNPs combined. (b) Forest plot showing the overall MR estimate leaving out one SNP at a time (All = All inverse variance weighted). (c) Scatter plot showing the association of each SNP with femoral neck BMD vs. the association with invasive mucinous EOC. (d) Funnel plot of instrument precision against the MR estimate for each variant.

BMD: bone mineral density; EOC: epithelial ovarian cancer; MR: Mendelian randomisation; SNP: single nucleotide polymorphisms.
